# Systematic Review of the Application of Perinatal Derivatives in Animal Models on Cutaneous Wound Healing

**DOI:** 10.3389/fbioe.2021.742858

**Published:** 2021-09-24

**Authors:** Melanie Pichlsberger, Urška Dragin Jerman, Hristina Obradović, Larisa Tratnjek, Ana Sofia Macedo, Francisca Mendes, Pedro Fonte, Anja Hoegler, Monika Sundl, Julia Fuchs, Andreina Schoeberlein, Mateja Erdani Kreft, Slavko Mojsilović, Ingrid Lang-Olip

**Affiliations:** ^1^ Division of Cell Biology, Histology and Embryology, Gottfried Schatz Research Center, Medical University of Graz, Graz, Austria; ^2^ Institute of Cell Biology, Faculty of Medicine, University of Ljubljana, Ljubljana, Slovenia; ^3^ Group for Hematology and Stem Cells, Institute for Medical Research, University of Belgrade, Belgrade, Serbia; ^4^ LAQV, REQUIMTE, Department of Chemical Sciences‐Applied Chemistry Lab, Faculty of Pharmacy, University of Porto, Porto, Portugal; ^5^ iBB–Institute for Bioengineering and Biosciences, Department of Bioengineering, Instituto Superior Técnico, Universidade de Lisboa, Lisboa, Portugal; ^6^ Associate Laboratory i4HB–Institute for Health and Bioeconomy at Instituto Superior Técnico, Universidade de Lisboa, Lisboa, Portugal; ^7^ Center for Marine Sciences (CCMar), Faculty of Sciences and Technology, University of Algarve, Faro, Portugal; ^8^ Department of Chemistry and Pharmacy, Faculty of Sciences and Technology, University of Algarve, Faro, Portugal; ^9^ Department of Obstetrics and Feto-maternal Medicine, Inselspital, Bern University Hospital, University of Bern, Bern, Switzerland; ^10^ Department for BioMedical Research (DBMR), University of Bern, Bern, Switzerland

**Keywords:** perinatal derivatives, placenta, cells, preclinical studies, animal models, wound healing, skin, cutaneous

## Abstract

Knowledge of the beneficial effects of perinatal derivatives (PnD) in wound healing goes back to the early 1900s when the human fetal amniotic membrane served as a biological dressing to treat burns and skin ulcerations. Since the twenty-first century, isolated cells from perinatal tissues and their secretomes have gained increasing scientific interest, as they can be obtained non-invasively, have anti-inflammatory, anti-cancer, and anti-fibrotic characteristics, and are immunologically tolerated *in vivo*. Many studies that apply PnD in pre-clinical cutaneous wound healing models show large variations in the choice of the animal species (e.g., large animals, rodents), the choice of diabetic or non-diabetic animals, the type of injury (full-thickness wounds, burns, radiation-induced wounds, skin flaps), the source and type of PnD (placenta, umbilical cord, fetal membranes, cells, secretomes, tissue extracts), the method of administration (topical application, intradermal/subcutaneous injection, intravenous or intraperitoneal injection, subcutaneous implantation), and the type of delivery systems (e.g., hydrogels, synthetic or natural biomaterials as carriers for transplanted cells, extracts or secretomes). This review provides a comprehensive and integrative overview of the application of PnD in wound healing to assess its efficacy in preclinical animal models. We highlight the advantages and limitations of the most commonly used animal models and evaluate the impact of the type of PnD, the route of administration, and the dose of cells/secretome application in correlation with the wound healing outcome. This review is a collaborative effort from the COST SPRINT Action (CA17116), which broadly aims at approaching consensus for different aspects of PnD research, such as providing inputs for future standards for the preclinical application of PnD in wound healing.

## Introduction

Human skin is the largest organ of the body that provides complex functions. It protects the body against mechanical, chemical, and physical impact, as well as dehydration. In addition, it modulates the body temperature and serves as a sensory organ. The compact part of the skin, the cutis, consists of the epidermis, a keratinized stratified squamous epithelium, and the underlying dermis, which is built from connective tissue. Below the cutis is the loosely-layered subcutis, which is enriched by adipose tissue.

Severe skin injury is the result of wounds caused by incision, excision, abrasion, burn, radiation or pressure. The degree of wounding describes the depth of damage to the respective skin layers. In first-degree wounds, only the epidermis is affected, in second-degree wounds, the epidermis and dermis are affected, and in third-degree wounds, all three skin layers including the subcutis are affected.

Skin damage is repaired by wound healing, a multistep, finely orchestrated process that includes hemostasis, inflammation, tissue growth (proliferation), and tissue remodeling (maturation) (Xue et al., 2018). This process is highly efficient in healthy individuals. However, the type, extent, and depth of injury, as well as any deviations in the fragile wound repair response, affect the healing outcome. Parameters such as aging, comorbidities (e.g., diabetes, obesity, arterial or venous insufficiencies, autoimmune diseases), and severe burn injuries constitute some of the causes that delay wound healing, often due to insufficient blood supply based on impaired wound revascularization (Demidova-Rice et al., 2012). Non-healing wounds that persist for more than 3 months are called *chronic wounds*. These types of wounds have failed to proceed through an orderly and timely reparative process to produce anatomic and functional integrity of the injured site (Lazarus et al., 1994) and are often detained in a self-perpetuating inflammatory stage that hinders progression to proliferation (Stojadinovic et al., 2008). Approximately 40 million patients worldwide suffer from chronic wounds, which are still a challenge to treat and constitute a significant financial burden on the health care system (Sen et al., 2009). Thus, new therapeutic approaches for wound healing are highly warranted.

A promising strategy for wound treatment is the application of human perinatal derivatives (PnD). PnD are birth-associated tissues such as the placenta and its annexes (human amniotic membrane (hAM), chorionic membrane, decidua, umbilical cord) and the amniotic fluid. PnD are an abundant source of extracellular matrix proteins, growth factors, and cells that have already been used in a wide variety of applications in tissue engineering and regenerative medicine (Deus et al., 2020). The application of the hAM has a long tradition in the treatment of wounds. Early studies from 1909 to 1913 reported successful use of the hAM in skin transplantation (Davis, 1909) and for treating burns and skin ulcerations as a biological dressing. Its use significantly reduced pain and increased the epithelialization rate of the traumatized skin without signs of infection (Sabella, 1913; Stern, 1913). Several clinical trials in the subsequent decades confirmed the successful use of the hAM for skin injuries (Silini et al., 2015). The twenty-first century brought the breakthrough for cell isolations from different placental regions and the progressive investigation of their therapeutic potential. Minimal criteria for the definition of PnD derived cells were described in 2008 (Parolini et al., 2008), and ongoing extensive characterization of PnD was performed (Caruso et al., 2012; Silini et al., 2020).

PnD derived cells have anti-inflammatory, immunomodulatory, anti-cancer, anti-fibrotic, anti-apoptotic, and anti-oxidant effects. They are immunologically tolerated *in vivo* and have been transplanted without signs of immunological rejection, meaning that the application does not require immunosuppressive treatment (Ueta et al., 2002; Bailo et al., 2004; Jirsova and Jones, 2017).

With the expanding knowledge that cells act by paracrine mechanisms, the released secretomes (cell-derived conditioned media, cell-derived extracellular vesicles) are gaining increasing interest. Placental cells secrete factors that are crucial for wound healing such as EGF, IL-8, and IGF-1, which modulate migration and proliferation of keratinocytes, fibroblasts, and endothelial cells (Kim et al., 2012). hAM mesenchymal stromal cells (hAMSC) and their secretomes exhibit beneficial, survival-enhancing effects on endothelial cells *in vitro* and were shown to stabilize endothelial networks in the Matrigel assay (König et al., 2012; König et al., 2015). Further, co-transplantation of hAMSC and placental endothelial cells enabled the formation of human capillaries connecting to the murine blood circulation in a mouse model *in vivo* (Kinzer et al., 2014).

As the formation of new blood vessels is critical for normal wound healing, innovations to improve wound revascularization could also lead to significant advances in wound healing therapy and patient care (Demidova-Rice et al., 2012). Improved techniques for tissue preservation, and recent advances in isolation and culture procedures for PnD derived cells paved the way for established clinical uses and investigative pre-clinical and clinical trials such as the application of placenta derived mesenchymal stromal-like cells (PDA-002) for the treatment of patients with diabetic foot ulcer wounds and peripheral arterial disease (Silini et al., 2015)^1^.

This review provides a thorough overview of the application of PnD in wound healing to assess its efficacy in preclinical animal models based on different wound types. We highlight the advantages and limitations of the most commonly used animal models and evaluate the impact of the type of PnD, the delivery method and the dose of cells/secretome application in correlation with the wound healing outcome.

## Methods and Search Strategy to Collect the Data

We performed a systematic literature search of the PubMed^®^ database covering a period from 2004 to 2020 and used the Boolean search string (Supplements) in accordance with the consensus of the scientific network of the COST SPRINT Action (CA17116) to identify articles exploring therapeutic options of PnD in *in vivo* experimental models of wound healing. Titles and abstracts were screened to select publications including *in vivo* models evaluating the efficacy of PnD. The search was limited to original research publications available as full text in English. The publications were cross-checked for meeting the inclusion criteria by an independent study. The selection workflow was compliant with the PRISMA guidelines (Page et al., 2021a; Page et al., 2021b).

We obtained 141 manuscripts investigating the application of PnD to animal wound- or angiogenic models. We further focused on studies of cutaneous wound healing, which used human PnD. Articles with insufficient data (insufficient group sizes, no statistics, no controls, no adequate figures) were excluded. After the screening process, we identified 79 relevant articles. We observed a continuous increase in publications on the application of PnD in animal models of cutaneous wound healing during the last decade. The major part of these studies was performed in Asia (China 41%, Iran 15%, rest of Asia 22%), followed by the United States and Canada (11%), Europe (6%), South America (4%) and Africa (Egypt 1%). From the relevant articles, we extracted data concerning animal species (rodents, large animals), diabetic status (diabetic or non-diabetic animals), the type of wounds (full-thickness wounds, burns, radiation-induced wounds, skin flaps, subcutaneous pockets), the source and type of PnD (placenta, umbilical cord, fetal membranes, cells, secretomes, tissue extracts), the method and dose of administration (topical application, intradermal/subcutaneous injection, intravenous or intraperitoneal injection, subcutaneous implantation) and the use of delivery systems (e.g., hydrogels, synthetic or natural biomaterials as carriers for transplanted cells, extracts or secretomes). Data extraction was performed to meet the populations, interventions, comparators, outcomes and study designs (PICOS) criteria (McKenzie et al., 2021).

## PnD Used for Cutaneous Wound Healing in Preclinical Studies

### Types, Dosage and Application Mode of PnD

**FIGURE 1:**
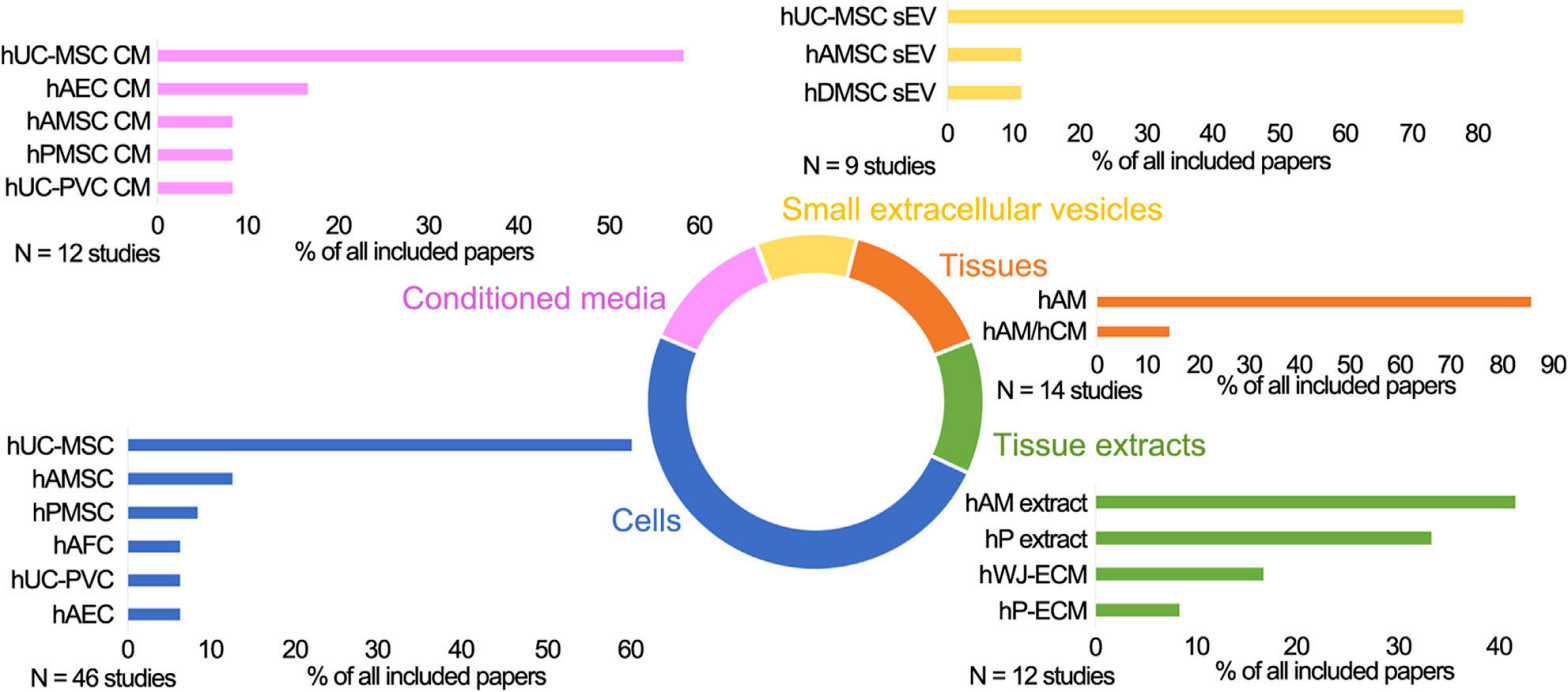
The schematic presentation of the perinatal derivatives used in skin wound healing in preclinical studies. Cells: hUC-MSC, human umbilical cord mesenchymal stromal cells; hAMSC, human amniotic membrane mesenchymal stromal cells; hPMSC, human placenta mesenchymal stromal cells; hUC-PVC, human umbilical cord perivascular cells; hAEC, human amniotic membrane epithelial cells; hAFSC, human amniotic fluid stem cells; hAFC, human amniotic fluid cells; Conditioned media (CM) derived from hUC-MSC, hAEC, hAMSC, hPMSC, hUC-PVC; Small extracellular vesicles (sEV) derived from hUC-MSC, hAMSC, hDMSC; Tissues: hAM, human amniotic membrane; hAM/hCM, human amniotic/chorionic membrane; Tissue extracts derived from hAM, hP (human placenta), hP-ECM (human placenta-derived extracellular matrix), hWJ-ECM (human Wharton’s jelly derived extracellular matrix).

In the papers included in this review, PnD were applied in the form of cells, cell secretomes (cell-derived conditioned media (CM), cell-derived small extracellular vesicles (sEV), tissue membranes and tissue extracts (Figure 1). Naming and abbreviations of the PnD types in the reviewed studies varied due to the authors’ discretion. To improve the comparability of data, we harmonized terms according to the recently published consensus nomenclature for perinatal tissues and cells (Silini et al., 2020). For example, as there is no consensus on the zones of Wharton’s jelly and on the experimental protocols for isolation of the cells thereof, we have used the single term *human umbilical cord mesenchymal stromal cells* (hUC-MSC), which comprises all potential cell subpopulations. The types and combinations of the PnD applied in these studies as well as the delivery systems are outlined in detail in Tables 1–5. Overall, in 14 of 79 studies, two different PnD types were addressed. The authors either compared the effect of different PnD (e.g., cells vs. cell-derived CM, cells vs. cell-derived sEV, cells vs. tissue), or two PnD types were applied in combination (different cell types, cells and cell-derived CM, cells and tissue).

**TABLE 1:** Application of perinatal cells in *in vivo* animal models of skin wound healing. Time points indicated in the “Outcome” column mean days (d), weeks (w) or hours (h) after treatment.

Perinatal cells					
PnD cell type	Dosage	Application (carrier)	Wound type, animal	Outcome	References
hAEC	3.54 × 10^6 cells/cm^2^	Intradermal injection	Full-thickness, diabetic mouse	hAEC showed higher engraftment, better keratinocyte-transdifferentiation rates and accelerated wound healing (wound closure and re-epithelialization) than hASC (d28)	Jin et al. (2016)
			Splint model		
hAFC, hAFSC	1.59 × 10^4 cells/cm^2^	Topical (polyester disks covered with collagen)	Full-thickness, rat	hAFC and hAFSC achieved accelerated wound closure, epithelization, collagen fiber production, angiogenesis and the disappearance of inflammatory cells compared to controls without cells (d14, d17, d21)	Yang et al. (2013)
hAFSC	6.41 × 10^6 cells/cm^2^	Intradermal/subcutaneous injection	Full-thickness, mouse	hAFSC showed a better keratinocyte-transdifferentiation and enhanced wound closure and early-stage repair of skin damage than fibroblasts and sham controls by creating a moderate inflammatory microenvironment (d21)	Sun et al. (2019)
		1.99 × 10^6 cells/cm^2^	Intradermal/subcutaneous injection	Full-thickness, mouse	hAFSC enhanced re-epithelialization and collagen III contents and achieved lower numbers of myofibroblasts and less fibrotic scarring than PBS controls (best effects d14, after d21 no significance). hAFSC engrafted in the epidermis and dermis	Fukutake et al. (2019)
hAMSC	3.54 × 10^6 cells/cm^2^	Intradermal injection	Full-thickness, diabetic mouse	hAMSC had higher engraftment and keratinocyte-transdifferentiation potential and induced a better healing potential (increased wound closure, re-epithelialization and cellularity) than ASC and fibroblasts (d7, d10 and d14)	Kim et al. (2012)
			Splint model		
	0.597 × 10^6 cells/cm^2^	Topical (Matrigel or Matriderm)	Full-thickness, mouse	In both application forms, hAMSC promoted neovascularization compared to control without cells. Matriderm/hAMSC enhanced wound closure (d8). Inhomogeneous distribution of Matrigel led to inadequate wound closure	Tuca et al. (2016)
		0.995 × 10^6 cells/cm^2^	Topical (Matriderm or PCL/PLA, with or without Matrigel)	Full-thickness, mouse	Matriderm and PCL/PLA were suitable as carriers for hAMSC. 3 days *in vitro* culture of scaffolds with hAMSCs without Matrigel before wound application is recommended. PCL/PLA showed higher cell adherence and counteract wound contracture (d14)	Vonbrunn et al. (2020)
	hUC-MSC	0.22 × 10^6 cells/cm^2^	Subcutaneous injection	Skin flap, mouse	hUC-MSC were mainly distributed in the subcutaneous flap tissues and increased survival of the flap, neovascularization and expression of bFGF and VEGF (d7)	Leng et al. (2012)
1.99 × 10^6 cells/cm^2^		Subcutaneous injection (SA/Col hydrogel)	Full-thickness, mouse	SA/Col hydrogel + hUC-MSC accelerated wound closure, formation of granulation, enhanced collagen deposition and angiogenesis. Hydrogel promoted the survival of hUC-MSC, enhanced growth factors secretion, and inhibited inflammation (d7, d14)	Zhang et al. (2021)
0.11 × 10^6 cells/cm^2^		Subcutaneous injection	Radiation, rat	hUC-MSC increased neovascularization and re-epithelization (d14, d21, d28)	Liu et al. (2018)
2.83 × 10^6 cells/cm^2^		Subcutaneous injection	Full-thickness, diabetic rat	Compared to un-transduced hUC-MSC, c-Jun overexpressing hUC-MSC accelerated wound closure, enhanced angiogenesis and re-epithelialization (d7, d10, d15, d17)	Yue et al. (2020)
1.27 × 10^6 cells/cm^2^		Topical (collagen-based scaffolds, Integra^®^ and Col)	Full-thickness, mouse	hUC-MSC accelerated angiogenesis compared to ASC and control without cells (d7, d10) and provided a suitable matrix for wound repair, without altering the inflammatory response in the animals	Edwards et al. (2014)
0.44 × 10^6 cells/cm^2^		Topical (collagen membrane)	Full-thickness, mouse	HOXA4-overexpressing hUC-MSC differentiated into epidermal cells and increased re-epithelialization of wounds and thickness of the epidermis (d7, d14, d21)	He et al. (2015)
Not specified		Topical (collagen membrane or collagen-fibrin membrane)	Full-thickness, mouse	hUC-MSC promoted wound healing. Collagen-fibrin carriers for hUC-MSC were more efficient in wound healing than collagen membrane carriers (d5, d10, d15)	Nan et al. (2015)
0.339 × 10^6 cells/cm^2^		Topical (fibrin-based scaffold)	Full-thickness, mouse	Fibrin-based scaffolds with hUC-MSC healed slowly with no scarring. Untreated wounds or wounds treated with scaffolds without cells healed rapidly but disorderly, due to wound retraction into scarring (d15, d21, d36)	Montanucci et al. (2017)
1.99 × 10^6 cells/cm^2^		Topical (PF-127/SAP hydrogel)	Full-thickness, mouse	PF-127/SAP hydrogel enhanced engraftment of hUC-MSC in the dermis and facilitated dermis regeneration (increase in thickness, collagen fibers, hair follicles), angiogenesis and M2 macrophage formation (d8)	Deng et al. (2020)
1.77 × 10^6 cells/cm^2^		Topical (collagen scaffold)	Full-thickness, diabetic mouse Splint model	Combination of hUC-MSC therapy and hyperbaric oxygen had a collaborative effect on wound-healing, with a faster healing rate compared to hUC-MSC alone (d7)	Pena-Villalobos et al. (2018)
0.39 × 10^6 cells/cm^2^		Topical (cell spray)	Burn 3rd degree, rat	hUC-MSC increased re-epithelialization compared to control without cells. hUC-MSC were detected in the burned areas at d7, d14, d21	Pourfath et al. (2018)
Not specified		Topical (collagen–chitosan laser drilling acellular dermal matrix)	Full-thickness, diabetic rat	hUC-MSC accelerated wound healing by activation of the Wnt signaling pathway (d7, d14, d21)	Han et al. (2019)
		200 cells/cm^2^	Topical (Integra^®^ collagen-based scaffold)	Burn full-thickness, pig	Low dose hUC-MSC regenerated wounds most efficaciously. Best effects were achieved by 40,000 cells/cm^2^ (accelerated epithelialization and vascularization, reduced signs of scarring, fibrosis, reduced numbers of macrophages compared to controls (d28)	Eylert et al. (2021)
		5,000 cells/cm^2^				
		40,000 cells/cm^2^				
		200,000 cells/cm^2^				
		400,000 cells/cm^2^				
	2000,000 cells/cm^2^					
	0.1 × 10^6 cells/cm^2^	Topical (PVA hydrogel membrane)	Non-healing skin lesions, dog	hUC-MSC induced a significant progress in skin regeneration with decreased extent of ulcerated areas	Ribeiro et al. (2014)
	29 × 10^6 cells/kg	Intravenous injection	Full-thickness, diabetic rat	UC-MSC were detectable in the wound tissue (d16). They improved wound healing by regulating inflammation, trans-differentiation and providing growth factors that promote angiogenesis, cell proliferation and collagen deposition (d8, d16)	Shi et al. (2020)
	25 × 10^6 cells/kg	Intravenous injection	Burn full-thickness, rat	hUC-MSC were detectable in the wound tissue for 3 weeks. They accelerated wound healing (wound closure, vascularization, collagen deposition) and decreased inflammation (w2, w3, w6, w8)	Liu et al. (2014)
	25 × 10^6 cells/kg	Intravenous injection	Burn full-thickness, rat	hUC-MSC attenuated burn-induced excessive inflammation via secretion of ant-inflammatory protein TSG-6 which inhibits activation of P38 and JNK signaling (6, 12, 24, 48 h)	Liu et al. (2016)
a) hUC-MSC-Fib	Not specified	Topical collagen-chitosan acellular dermal matrix - tissue engineered dermis (TED)	Full-thickness, pig	me-VEGF-hUC-MSC-Fib improved the vascularization of tissue-engineered dermis and induced a higher wound healing than controls (me-hUC-MSC, empty capsule and PBS-treated group) (w3)	Han et al. (2014)
b) me-VEGF- hUC-MSC-Fib					
a) hUC-MSC	5.09 × 10^6 cells/cm^2^	(a-b) Intradermal injection	Full-thickness, mouse	Transplantation of celecoxib (anti-inflammatory drug) -preconditioned hUC-MSC-End showed higher wound healing potential than hUC-MSC and hUC-MSC-End (d7)	Kaushik and Das, (2019)
b) hU-MSC-End			Splint model		
hPMSC	45 × 10^6 cells/kg	Intraperitoneal and intradermal injection	Full-thickness, mouse	hPMSC enhanced wound healing through release of proangiogenic factors and decreased proinflammatory cytokines. The intraperitoneal injections are more effective than intradermal injections (d7)	Abd-Allah et al. (2015)
	1.99 × 10^6 cells/cm^2^	Subcutaneous injection	Full-thickness, diabetic rat	hPMSC accelerated wound closure, increased collagen deposition, granulation tissue and epidermis thickness (d15). Wound healing was accelerated by decrease of local pro-inflammatory cytokines TNF-α, IL-6 and IL-1, increase of anti-inflammatory IL-10	Wang et al. (2016)
		5.09 × 10^6 cells/cm^2^	Topical (Matrigel)	Full-thickness, mouse	PDGFR-β- hPMSC displayed a superior angiogenic property and exerted enhanced therapeutic efficacy on cutaneous wound healing compared to PDGFR-β -negative hPMSC (d7, d14)	Wang et al. (2018)
				Splint model		
hUC-PVC	7.96 × 10^6 cells/cm^2^	Topical (fibrin gel)	Full-thickness, mouse	hUC-PVC accelerated re-epithelization and dermal repair and wound strength compared to treatment without cells (d7)	Zebardast et al. (2010)
		1.27 × 10^6 cells/cm^2^	Topical (decellularized dermal matrix scaffold)	Full-thickness, diabetic rat	hUC-PVC accelerated wound closure rate (faster re-epithelization, more granulation tissue formation, decreased scarring and increased neovascularization than treatment without cells (d14, d21)	Milan et al. (2016)
a) hAEC-Ker	a) 7,500 cells/cm^2^	Topical (plasma-based gel)	Burn 2nd degree, full-thickness excision, rat	Scaffolds seeded with hUC-MSC-Fib and hAEC-improved re-epithelization concurrent with reduced apoptosis compared to treatment without cells (d10, d20)	Mahmood et al. (2019)
+ b) hUC-MSC-Fib	b) 6,250 cells/cm^2^				
a) hAMSC	0.6 × 10^6 cells/cm^2^	(a-c) Topical (Matriderm)	Full thickness, mouse	hAMSC, hCP-MSC-bv and hUC-MSC induced faster wound healing and vascularization compared to controls without cell treatment (d8). hCP-EC co-application did not further improve the advantageous effects of MSC.	Ertl et al. (2018)
b) hCP-MSC-bv					
c) hUC-MSC					
+co-application with hCP-EC					

Abbreviations: bFGF, basic fibroblast growth factor; coI, collagen type I; hAEC, human amniotic membrane epithelial cells; hAEC-Ker, human amniotic membrane epithelial cells derived keratinocytes; hAFC, human amniotic fluid cells; hAFSC, human amniotic fluid stem cells; hAMSC, human amniotic membrane mesenchymal stromal cells; hASC, human adipose mesenchymal stromal cells; hCP-EC, human chorionic plate endothelial cells; hCP-MSC-bv, human chorionic plate mesenchymal stromal cells derived from blood vessels; hPMSC, human placenta mesenchymal stromal cells; hUC-MSC, human umbilical cord mesenchymal stromal cells; hU-MSC-End, human umbilical cord mesenchymal stromal cells -endothelial transdifferentiated; hUC-MSC-Fib, human umbilical cord mesenchymal stromal cells derived fibroblasts; hUC-PVC, human umbilical cord perivascular cells; me-VEGF- hUC-MSC-Fib, microencapsulated VEGF-gene modified human umbilical cord mesenchymal stromal cells derived fibroblasts; PCL/PLA, Poly(caprolactone)/poly(l-lactide); PDGFA, platelet derived growth factor A; PDGFR-β, platelet derived growth factor receptor β; PF-127/SAP, Pluronic F-127 hydrogel plus antioxidant sodium ascorbyl phosphate; PVA, polyvinyl alcohol; SA, sodium alginate; TED, Topical collagen-chitosan acellular dermal matrix - tissue engineered dermis; TNF-α, tumor necrosis factor α; TSG-6, TNF-stimulated gene 6 protein; VEGF, vascular endothelial cell growth factor; IL-1, IL-6, IL-10, interleukin-1, -6, -10.

**TABLE 2:** Application of perinatal cell-conditioned medium (CM) alone or compared to/combined with perinatal cells in *in vivo* animal models of skin wound healing. Time points indicated in the “Dosage” and Outcome” columns mean days (d), weeks (w) or hours (h) of/after treatment.

Perinatal cell-conditioned medium (CM)					
PnD	Dosage	Application (carrier)	Wound type, animal	Outcome	References
hAEC-CM	100 μl (48 h) CM/cm^2^ on d1 and d3	Subcutaneous injection	Full-thickness, mouse	hAEC-CM enhanced wound healing (closure, tissue reorganization, replacement of skin appendages), whereas CM+ ERK, JNK and AKT- inhibitors impaired wound healing (d7, d14). Control mice received PBS injection	Zhao et al. (2016)
hPMSC-CM	31.85 μl (72 h) CM/cm^2^	Subcutaneous injection	Burn degree n.d., mouse	hPMSC were maintained in normoxic or hypoxic conditions. Hypoxic CM reduced scar formation, while there was no marked difference between normoxic CM and controls (normal medium) at d8	Du et al. (2016)
hUC-MSC-CM	50 μl (5 μg/ml) (48 h) CM/cm^2^ every 2nd d for 8w	Topical (hydrogel)	Radiation, rat	Hydrogel containing hUC-MSC-CM accelerated wound closure, sebaceous gland cell-like regeneration and angiogenesis compared to EGF gel and negative control (w2, w4, w6, w8, wound treatment every 2 days)	Sun et al. (2019)
	(48 h) CM (volume not specified)	Topical (SA/gelatin hydrogel)	Full-thickness, rat	Hydrogel containing CM of UC-MSC transfected cells accelerated wound contraction and promoted neovascularization, skin-appendages, epithelialization compared to control (PBS or Hydrogel treatment without CM (d14)	Sabzevari et al. (2020)
			Splint model		
	**Perinatal cell-conditioned medium (CM) compared to/or combined with perinatal cells**					
**PnD**	**Dosage (CM harvesting time)**	**Application (carrier)**	**Wound type, animal**	**Outcome**	**References**
a) hAMSC	2.546 × 10^6 cells/cm^2^	Subcutaneous injection	Burn 2nd degree, mouse	hAMSC and hAMSC-CM similarly accelerated re-epithelialization and cell proliferation compared to controls without cells or CM (d7, d14, d21), increased expression of CK19 and PCNA, inhibited heat stress-induced apoptosis through activating PI3K/AKT signaling pathway	Li et al. (2019)
b) hAMSC-CM	254.6 μl (48 h) CM/cm^2^				
a) hAEC-CM	I exp. 7 μl (72 h) CM/cm^2^ d1, d7	Topical (cell spray)	Burn partial-thickness, guinea pig	hAEC, hAEC-CM, or the combination of both improved epithelialization compared to controls without cell or CM treatment (d7 – d21). Frequent application of hAEC-CM for every day achieved better results than 2-fold application at d0 and d7	Payne et al. (2010)
b) hAEC + hAE-CM	33,333 cells/cm^2^ d1, d7				
c) hAEC + unconditioned medium	II exp. 10 μl (72 h) CM/cm^2^ on every 2nd d or 4th d or 7th d for 3w				
a) hUC-MSC	7.07 × 10^6 cells/cm^2^	(a-b) Subcutaneous injection	Full-thickness, diabetic mouse	hUC-MSC and hUC-MSC-CM accelerated wound closure and angiogenesis, with similar effects at d10. CM induced better effects in wound healing and higher expression of PDGFß and KGF in wounds at d14	Shrestha et al. (2013)
b) hUC-MSC-CM	212.3 μl (24 h) CM/cm^2^				
a) hUC-MSC or hU-MSC-End	1.77 × 10^6 cells/cm^2^	(a-b) Intradermal injection	Full-thickness, mouse Splint model	hUC-MSC and CM accelerated wound closure, regeneration capacity and neovascularization. hUC-MSC-End achieved better cellular and paracrine effects than hUC-MSC (d7, d12). Effects of cells were not directly compared to the effects of CM.	Aguilera et al. (2014)
b) hUC-MSC-CM or hU-MSC-End-CM	212.3 μl (48 h) CM/cm^2^				
a) hUC-MSC	1.99 × 10^6 cells/cm^2^	(a-b) Subcutaneous injection	Full-thickness, diabetic mouse	hUC-MSC and hUC-MSC-CM similarly improved angiogenesis, re-epithelialization and granulation (d14). Fibroblasts or PBS served as controls	Zhang et al. (2020)
b) hUC-MSC-CM	298 μl (24 h) CM/cm^2^				
a) hUC-MSC	1.99 × 10^6 cells/cm^2^ 3.54 × 10^6 cells/cm^2^ 199 μl (72 h) CM/cm^2^ 354 μl (72 h) CM/cm^2^	(a-b) Topical (AV/PCL scaffold) or subcutaneous injection	Full-thickness, diabetic mouse	hUC-MSC and hUC-MSC-CM applied by AV/PCL carrier or subcutaneous injection similarly achieved better effects on wound healing (re-epithelialization, collagen deposition, angiogenesis and immunomodulation) than controls (fibroblasts, fibroblast-CM, unconditioned medium (d7, d14, d28)	Raj et al. (2019)
b) hUC-MSC-CM					
a) hUC-MSC	6.37 × 10^4 cells/cm^2^	Topical (alginate gel) with cells or CM	Full-thickness, mouse Splint model	hUC-MSC-alginate and hUC-MSC-CM-alginate achieved faster wound healing than control groups (FBS-alginate, PBS-alginate), (d10, d15)	Wang et al. (2016)
b) hUC-MSC-CM					
a) hUC-PVC	3.54 × 10^6 cells/cm^2^	(a-b) Intradermal injection combined with topical application	Full-thickness, mouse Splint model	hUC-PVC and hUC-PVC-CM accelerated wound closure and healing (collagen deposition and angiogenesis) compared to fibroblasts and fibroblast-CM (d4, d7, d14). Effects of cells were not directly compared to the effects of CM.	Shohara et al. (2012)
b) hUC-PVC- CM	354 μl (48 h) CM/cm^2^				

Abbreviations AV/PCL, Aloe vera/po lycaprolactone; hAEC, human amniotic membrane epithelial cells; hAMSC, human amniotic membrane mesenchymal stromal cells; hPMSC, human placenta mesenchymal stromal cells; hUC-MSC, human umbilical cord mesenchymal stromal cells; hU-MSC-End, human umbilical cord mesenchymal stromal cells-endothelial transdifferentiated; hUC-PVC, human umbilical cord perivascular cells; CM, conditioned medium derived from hAEC, hAMSC, hPMSC, hUC-MSC, hUC-MSC-End, hUC-PVC; SA, sodium alginate.

**TABLE 3:** Application of perinatal cell-derived small extracellular vesicles (sEV) alone or compared to perinatal cells in *in vivo* animal models of skin wound healing. Time points indicated in the “Dosage” and “Outcome” columns mean days (d) or weeks (w) of/after treatment.

Perinatal cell-derived small extracellular vesicles (sEV)					
PnD	Dosage	Application (carrier)	Wound type, animal	Outcome	References
hUC-MSC-sEV	100 μg/cm^2^	Subcutaneous injection	Burn 2nd degree, rat	hUC-MSC-sEV enhanced re-epithelization and promoted self-regulation of Wnt/β-catenin signaling during the tissue remodeling period of cutaneous regeneration compared to PBS control (w2, w4)	Zhang et al. (2016)
	100 μg/cm^2^	Subcutaneous injection	Burn 2nd degree, rat	hUC-MSC-sEV promoted re-epithelization and angiogenesis compared to controls (PBS, fibroblast-Ex, d7). Proangiogenic effects were inhibited by interference of Wnt4 expression in hUC-MSC- sEV.	Zhang et al. (2015b)
	318 μg/cm^2^	Subcutaneous injection	Burn 2nd degree, rat	hUC-MSC-sEV accelerated wound closure and angiogenesis compared to control (PBS, d13). Overexpression of Ang-2 in hUC-MSC-sEV further enhanced therapeutic effects. Knockdown of Ang-2 in hUC-MSC-sEV abrogated these effects	(Liu et al., 2020)
	127 µg/cm^2^	Subcutaneous injection	Burn 2nd degree, mouse	hUC-MSC- sEV treated with blue light (455 nm) achieved better angiogenic effects than untreated hUC-MSC- sEV (d7)	Yang et al. (2019)
	127 µg/cm^2^	Topical injection (Pluronic F127 hydrogel)	Full-thickness, diabetic rat	hUC-MSC-sEV -hydrogel and hUC-MSC- sEV -PBS accelerated the wound closure rate and vascularization compared to controls (gel, PBS). hUC-MSC- sEV -hydrogel achieved better effects than hUC-MSC- sEV -PBS (d7, d10, d14)	Yang et al. (2020)
	hDMSC-sEV	2.6 × 10^10 particles/cm^2^ on every 7th d for 4w	Subcutaneous injection	Full-thickness, diabetic mouse	hDMSC-sEV accelerated wound closure and collagen deposition compared to PBS controls (d14, d21)	Bian et al. (2020)
	**Perinatal cell-derived small extracellular vesicles (sEV) compared to perinatal cells**					
	**PnD**	**Dosage**	**Application (carrier)**	**Wound type, animal**	**Outcome**	**References**
	a) hAMSC	2,222; 22,222; or 222,222 cells/cm^2^ Dose of sEV not specified	a) Subcutaneous injection (collagen) (b-c) Topical (collagen)	Full-thickness, rat	hAMSC and hAMSC-sEV enhanced wound closure and epidermalization. hAMSC-miR-135a-sEV induced faster wound healing than hAMSC-sEV (d5). Higher cell dose achieved better results than lower cell dose	Gao et al. (2020)
b) hAMSC-sEV						
c) hAMSC-miR-135a-sEV						
a) hUC-MSC	1.56 × 10^6 cells/cm^2^	(a-b) Subcutaneous injection	Full-thickness, mouse	hUC-MSC-sEV attenuated full-thickness skin wounds by enhancing epidermal re-epithelialization and dermal angiogenesis compared to hUC-MSC (d7, d14)	Zhao et al. (2020)
b) hUC-MSC-sEV	156 μg-sEV/cm^2^				
a) hUC-MSC-sEV	100 μg sEV/cm^2^	Subcutaneous injection	Burn 2nd degree, rat	hUC-MSC and hUC-MSC-sEV similarly accelerated re-epithelialization and increased expression of CK19, PCNA, and collagen I compared to control (PBS, fibroblast, Fibroblast-sEV, d7, d14) via Wnt4 pathway. hUC-MSC-sEV reduced heat stress-induced apoptosis via activation of AKT pathway	Zhang et al. (2015a)
b) hUC-MSC	0.5 × 10^6 cells/cm^2^				

Abbreviations; hAMSC, human amniotic membrane mesenchymal stromal cells; hAMSC-miR-135a, human amniotic membrane mesenchymal stromal cells overexpressing miR-135a; hDMSC, human decidua mesenchymal stromal cells; hUC-MSC, human umbilical cord mesenchymal stromal cells; sEV, small extracellular vesicles derived from hAMSC, hDMSC, hUC-MSC; miR-135a-sEV, sEV overexpressing microRNA135a.

**TABLE 4:** Application of perinatal tissues alone or compared to/combined with perinatal cells or cells of non-perinatal origin in *in vivo* animal models of skin wound healing. Stated time points in the “Outcome” column mean days (d) or weeks (w) after treatment.

Perinatal tissues					
PnD		Carrier of the topical PnD application	Wound type, animal	Outcome	References
a) hAM	wa 0.2 cm^2^	Topical	Full-thickness, mouse Splint model	hAM-silk fibroin scaffolds achieved better epidermal and dermal regeneration than hAM-treated and untreated wounds (d30)	Arasteh et al. (2020)
b) hAM + silk fibroin					
a) hAM	wa 1.77 cm^2^	Topical	Full-thickness, diabetic rat, non-diabetic control rat	hAM-S seemed to have better effects on the healing of diabetic wounds than hAM (d7, d14, d21)	Nasiry et al. (2020)
b) hAM-S			Splint model		
hAM	wa 1.69 cm^2^	Topical	Full-thickness, rat	hAM promoted wound closure (d3, d5, d7), and enhanced VEGF and α- SMA expression (d7). Reduced TGF-β1 expression at an early stage (d3) alleviated wound inflammation, promoted tissue regeneration and relieved scar formation compared to PBS treated wounds	Song et al. (2017)
Dehydrated hAM/chorion (EpiFix^®^)	Not applicable	Subcutaneous implantation	Subcutaneous pocket model, mouse	hAM/chorion implants recruited more mesenchymal progenitor cells to the site of implantation compared to normal skin and the sham implant site (d7)	Koob et al. (2013)
Dehydrated hAM/chorion (EpiFix^®^)	Not applicable	Subcutaneous implantation	Subcutaneous pocket model, mouse	hAM/chorion implants displayed a steady increase in microvessels approaching that of healthy and healing skin (d28)	Koob et al. (2014)
**Perinatal tissues compared to/or combined with perinatal cells**					
**PnD**	**Dosage**	**Application**	**Wound type, animal**	**Outcome**	**References**
a) hUC-MSC	1 × 10^6 cells/cm^2^ wa 1 cm^2^	a) Subcutaneous injection	Full-thickness, mouse	Combination of hUC-MSC and hAM achieved better wound healing (reduced scar formation with hair growth and improved biomechanical properties of regenerated skin) than hUC-MSC alone (d14) and untreated wounds	Sabapathy et al. (2014)
b) hUC-MSC+ hAM		b) Topical			
c) hAM					
a) hUC-MSC	0.7 × 10^6 cells/cm^2^ wa 1.54 cm^2^	Subcutaneous injection or topical (b-c) Topical	Burn 3rd degree, rat	hUC-MSC/hAM combination induced better wound healing (re-epithelialization, formation of granulation tissue, and hemorrhage) than hAM and hUC-MSC alone (d14)	Hashemi et al. (2020)
b) hAM					
c) hUC-MSC+hAM					
**Perinatal tissues combined with cells of non-perinatal origin**					
**PnD**		**Carrier of the topical PnD application**	**Wound type, animal**	**Outcome**	**References**
a) hAM	wa 11–18 cm^2^ 0.5 × 10^6 cells/cm^2^	Topical	Burn 3rd degree, rat	hAM seeded with fibroblasts or with ASC similarly showed better wound healing than hAM-only and control (Vaseline gauze) (d7, d14, d20, d28, d40)	Motamed et al. (2017)
b) hAM+hFib					
c) hAM+hASC					
a) hAM	wa 0.79 cm^2^ 10,000 cells/cm^2^	a) Topical (b-c) Topical silk fibroin	Burn 3rd degree, mouse	Silk fibroin accelerated wound healing compared to hAM only. hAM/silk fibroin+hASC achieved better effects than hAM/silk fibroin without ASC (d7, d14, d28) and reduced post burn scars	Gholipourmalekabadi et al. (2018)
b) hAM/silk fibroin					
c) hAM/silk fibroin+hASC					
hAM (loaded or injected with autologous or allologous rabbit BM-MSC	wa 5.06 cm^2^	Topical	Full-thickness, rabbit	hAM grafts loaded with autologous and allologous BM-MSC similarly accelerated wound closure compared to hAM grafts with injected BM-MSC (d7, d12, d15)	Kim et al. (2009)
	88,888 cells/cm^2^ on hAM				
	3.02 × 10^6 cells/cm^2^ i.d				
a) hAM	wa 2 cm^2^ number of cells per cm^2^ Not specified	Topical	Radiation followed by full-thickness, rat	hAM+BM-MSC and hAM+ freeze-dried BM-MSC similarly accelerated wound closure compared to hAM only. Inflammation and exudations were absent when hAM was used in contrast to non-treated wounds (Observation period 90 days)	Kakabadze et al. (2019)
b) hAM+BM-MSC					
c) hAM+ freeze-dried rat BM-MSC					
a) Dehydrated hAM (Amniofix ^®^)	wa 4 cm^2^ 0.1 × 10^6 cells/cm^2^	Topical	Full-thickness, rat	All treatments resulted in similar wound closure (d7-d21). TGF-β3 expressing cells decreased the scar formation (d85)	Samadikuchaksaraei et al. (2016)
b) Amniofix ^®^ +BM-MSC					
c) Amniofix ^®^ +TGFβ3 expressing BM-MSC					
a) hAM	wa 2.25 cm^2^	Topical	Full-thickness, rat	Men-MSC+hAM improved wound closure, angiogenesis and re-epithelization compared to hAM-only (d14)	Farzamfar et al. (2018)
b) hAM+ Men-MSC	30,000 cells/cm^2^				

Abbreviations: α-SMA, alpha-smooth muscle actin; BM-MSC, bone-marrow mesenchymal stromal cells; hAM, human amniotic membrane; hAM-S, bioengineered 3D hAM-scaffold; hASC, human adipose mesenchymal stromal cells; hFib, human fibroblasts; hUC-MSC, human umbilical cord mesenchymal stromal cells; Men-MSC, menstrual blood mesenchymal stromal cells; TGF-β1, transforming growth factor beta-1; VEGF, vascular endothelial cell growth factor; wa, wound area covered by tissue membranes.

**TABLE 5:** Application of perinatal tissue extract alone or compared to/combined with with perinatal tissue or cells of non-perinatal origin in *in vivo* animal models of skin wound healing. Time points indicated in the “Dosage” and “Outcome” column mean days (d) or weeks (w) of/after treatment.

Perinatal tissue extracts					
PnD	Dosage	Application (carrier)	Wound type, animal	Outcome	References
hAM extract	83.3 μg extract/cm^2^	a) Topical	Burn 1st degree, rat	hAM+chitosan-gel increased cutaneous regeneration (granulation tissue, fibroblast proliferation, vascularization) compared to controls (PBS, gel) (d15, d25, d31). High concentrated hAM extract (1 mg/ml) achieved better effects than low concentrated hAM extract (0.1 mg/ml)	Momeni et al. (2018)
	8.33 μg extract/cm^2^	b) Topical (chitosan hydrogel)			
hAM extract	100 μg/ml wound dressing (applied volume not spec.)	Topical (PVA/SA gel)	Full-thickness, rat	hAM+PVA/SA-gel accelerated wound closure, increased re-epithelization, granulation tissue areas, neovascularization, collagen proliferation and reduced number of inflammatory cells compared to Medifoam™ hydrogel or the control (sterile gauze) (d6, d9, d12)	Choi et al. (2014)
hP extract	3.98 µl extract/cm^2^	Subcutaneous injection	Full-thickness, mouse	hP extract accelerated wound healing (d3-d9) TGF-β increased in the early phase of wound healing (d6) and VEGF in the late phase (d14) compared to PBS control	Hong et al. (2010)
	3.33 μl extract/cm^2^/d	Subcutaneous injection or Intraperitoneal injection	Skin flap, rat	hP extract enhanced flap survival, angiogenesis, reduced necrotic areas, induced antioxidative response and inhibited apoptosis compared to PBS control. Daily application (d0-d6) of low dose localized or systemic hP injections or high dose. Systemic high dose HP injections showed the best effects. (d7)	Kwon et al. (2019)
	0.45 and 1.35 ml/kg/d every day for 7 days				
a) hP extract	a) 35 μg/cm^2^	Subcutaneous injection	Full-thickness, rat	hP and placental laminin accelerated wound closure compared to PBS controls (d5, d7, d9)	Mukherjee et al. (2020)
b) Placental laminin	b) 11 μg/cm^2^				
a) hAM powder	2 μg hAM/cm^2^	Topical	Burn 2nd degree, rat	hAM powder, AV, and hAM+AV accelerated wound healing compared to untreated wounds (d24)	Rahman et al. (2019)
b) hAM powder+AV	1 μg hAM + 1 μg AV/cm^2^				
Solubilized hAM	Not specified	Topical (hyaluronic acid hydrogel)	Full-thickness, mouse	hAM-hyaluronic acid hydrogel accelerated wound closure, wound re-epithelialization, vascularization compared to controls (hydrogel, untreated). Hydrogel ± hAM counteracted wound contracture in contrast to untreated wounds (d7, d14)	Murphy et al. (2017)
hP–ECM	Not specified	Topical (hybrid with silk fibroin)	Full-thickness, rat	hP-ECM-silk fibroin scaffolds accelerated wound closure (pronounced angiogenesis, enhanced granulation tissue formation, early re-epithelialization) compared to controls (collagen-silk-fibroin scaffolds, sham) (d7, d14, d21)	Rameshbabu et al. (2018)
hWJ-ECM	450 μl/cm^2^	Topical (hWJ-ECM scaffold)	Full-thickness, mouse	WJ-ECM scaffolds accelerated wound closing and re-epithelization compared to wounds without scaffolds (d7, d12) and counteract wound contracture (d18)	Beiki et al. (2017)
hWJ-ECM	353.7 μl/cm^2^	Topical (Matrigel)	Full-thickness, mouse	Acellular hUC-WJ+Matrigel enhanced wound closure and augmented the differentiation of fibroblasts into myofibroblasts compared to control (DMEM-Matrigel) (d5, d7)	Bakhtyar et al. (2017)
**Perinatal tissue extracts compared to perinatal tissue**					
PnD	**Dosage**	**Application (carrier)**	**Wound type, animal**	**Outcome**	**References**
a) hAM powder	Not specified	Topical	Full-thickness, pig	Amnion+hydrogel and amnion powder accelerated wound healing compared to Amniograft^R^ > hydrogel only > untreated wounds > graft jacket. The treatment with graft jacket-only led to the worst healing (most wound, most contraction, least epithelialization (d28)	Murphy et al. (2020)
b) hAM powder+hydrogel					
c) Amniograft^R^					
**Perinatal tissue extracts combined with cells of non-perinatal origin**					
**PnD**	**Dosage**	**Application (carrier)**	**Wound type, animal**	**Outcome**	**References**
a) hPE+autologous BM-MSC	Concentration of hPE not specified (commercial preparation) 1 × 10^6 cells/cm^2^	Topical	Full-thickness, rabbit	hP-E+ BMSC achieved better results on wound healing (accelerated wound closure, earlier disappearance of inflammatory reaction, better epithelialization, neovascularisation, and collagen formation than the other groups (d7, d14, d21, d30)	Akela et al. (2013)
b) hPE+buffy coat in autologous plasma)					
c) hPE+autologous plasma					

Abbreviations: AV, aloe vera; BM-MSC, bone marrow mesenchymal stromal cells; hAM, human amniotic membrane; hPE, human placenta extract; hP-ECM, human placenta extracellular matrix; hUC-WJ-ECM, human umbilical cord Wharton´s jelly extracellular matrix; PVA, polyvinyl alcohol; SA, sodium alginate.

To standardize the different dosages of PnD used in the reviewed studies, the doses are presented as the number, weight (μg), or volume (μl) of the specific PnD per wound area (cm^2^), when the PnD were administered locally (topically, intradermally/subcutaneously), or per body weight (kg) of an animal, when PnD were applied systemically (intravenously or intraperitoneally). To determine the dose of locally administered PnD per cm^2^, we divided the quantity of applied PnD by the wound area. In most of the studies, both the wound area and the exact quantity of PnD were indicated.

#### Perinatal Cells

Perinatal cells were the most commonly used PnD type in preclinical studies of cutaneous wound healing (49.5%) (Table 1). Mostly, hUC-MSC were used (60.4%), followed by hAMSC (12.5%) and MSC isolated from the whole placenta (hPMSC, 8.3%). Cells were applied as suspensions injected intradermally/subcutaneously or intravenously, topically by cell spraying, or seeded on electrospun or biological scaffolds, including the amniotic membrane. Cell therapy implemented by administering cells to the bloodstream may lead to the accumulation of injected cells in other organs such as the lungs (Ankrum and Karp, 2010).

**FIGURE 2:**
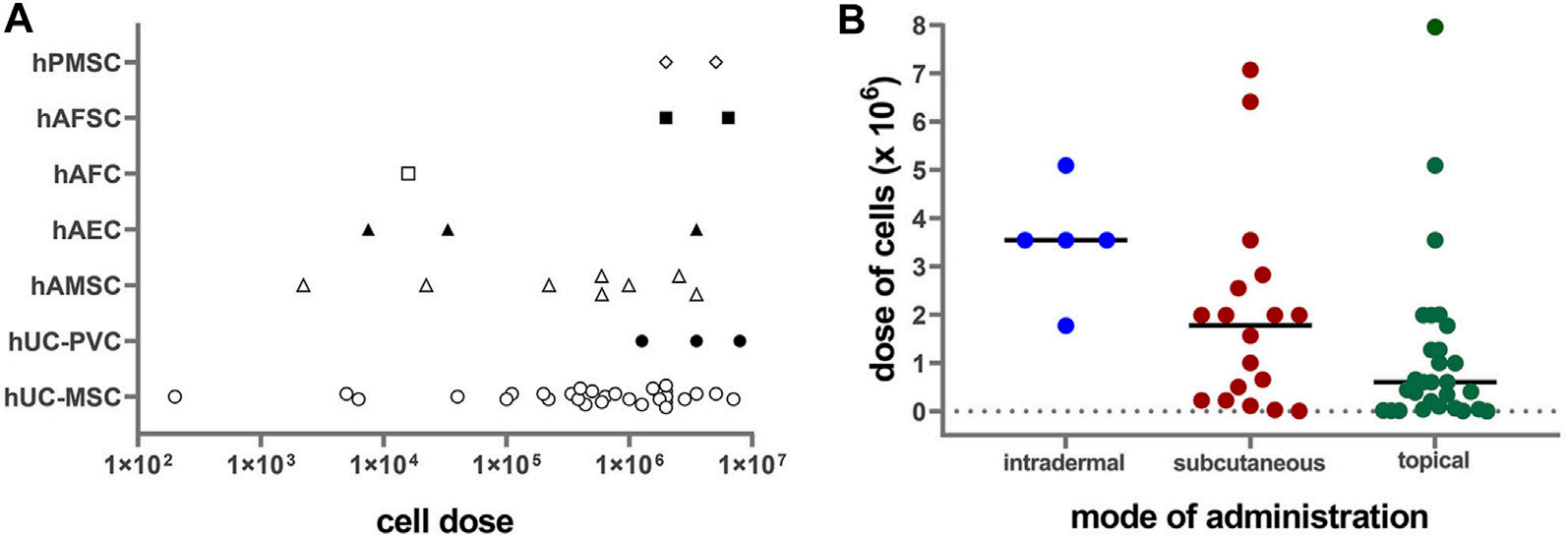
**(A)** Correlation of applied PnD cell type versus cell number/cm^2^ on a logarithmic scale (Log10). hUC-MSC, human umbilical cord mesenchymal stromal cells; hAMSC, human amniotic membrane mesenchymal stromal cells; hPMSC, human placenta mesenchymal stromal cells; hAFC, human amniotic fluid cells; hAFSC, human amniotic fluid cells; hUC-PVC, human umbilical cord perivascular cells; hAEC, human amniotic membrane epithelial cells. **(B)** Correlation of cell doses with the mode of administration. Lines represent median values.

Perinatal cells were applied in doses ranging from 200 cells per cm^2^ up to 8 million cells per cm^2^ (Figure 2A). The most frequently applied dose was around 2 million cells per cm^2^ (mode = 1.99 × 10^6^/cm^2^, median = 1 × 10^6^/cm^2^). Regarding mode of application, lower doses of cells were used for topical application (median = 0.6 × 10^6^/cm^2^) than for subcutaneous (median = 1.78 × 10^6^/cm^2^) and intradermal (median = 3.54 × 10^6^/cm^2^) applications (Figure 2B). Mostly used dose for full-thickness wound model was 1.99 × 10^6^/cm^2^ (median = 1.88 × 10^6^/cm^2^), while for the other wound models the doses were usually less than 1 × 10^6^/cm^2^ (median = 0.12 × 10^6^/cm^2^ for burn wounds, 0.11 × 10^6^/cm^2^ for radiation wounds, 0.22 × 10^6^/cm^2^ for skin flaps, and 0.1 × 10^6^/cm^2^ for non-healing skin lesions). The median cell doses for mice were higher (1.99 × 10^6^/cm^2^) than those for the rats (0.16 × 10^6^/cm^2^). The systemic doses were 25 × 10^6^/kg and 29 × 10^6^/kg when applied intravenously, and 45 × 10^6^/kg, when applied intraperitoneally (Liu et al., 2014; Abd-Allah et al., 2015; Liu et al., 2016; Shi et al., 2020). Moreover, few studies compared the effect of applying different cell concentrations. Namely, in a full-thickness skin wound model on rats, increasing the number of hAMSC injected into the wound bed from 2 × 10^3^/cm^2^ to 2 × 10^5^ cells/cm^2^, enhanced the wound healing results (Gao et al., 2020). On the other hand, in a full-thickness burn wound model on pigs, low doses, ranging from 200 to 40,000 cells per cm^2^, topically applied in combination with a collagen-based scaffold, demonstrated superior wound healing of full-thickness burn wounds, compared to higher doses (2 × 10^5^/cm^2^ to 2 × 10^6^/cm^2^) and controls (Eylert et al., 2021). In a study that compared systemic (intraperitoneal) and local (intradermal) application, the intraperitoneal injections of hPMSC showed better results than the local one, in terms of histological scores, expression of the healing promoting factors, as well as the engraftment into the wounded skin (Abd-Allah et al., 2015).

#### Perinatal Cell-Derived Conditioned Medium (CM)

Of the studies included in this review, 12.9% used perinatal cell-derived CM (Table 2). The CM derived from hUC-MSC was the most commonly used CM (58.3%). The CM is a medium collected from cell cultures and contains various cell products released by cells. These products may be proteins, non-coding RNAs, growth factors, antioxidants, proteasomes, and extracellular vesicles, which vary depending on the cell type and cell culture conditions (Maguire, 2013). The advantage of using CM collected from MSC to study regeneration lies in the ease of medium availability, storing, freezing, drying, and transporting, as well as in its lack of immunogenicity (Gunawardena et al., 2019).

CM was usually collected after 24, 48, and 72 h of cell cultivation. It was mainly applied intradermally/subcutaneously or topically and in combination with hydrogels or as a cell spray. Doses of the CM used ranged from 7 to 354 μl/cm^2^. No studies examined the effects of different CM dosages on the efficacy of wound healing.

Several wound healing studies have investigated the combined treatment of cell-derived CM and corresponding cells, or have compared the efficacy of cell-derived CM versus cells alone. While both CM and its corresponding perinatal cells, alone or in combination, showed an increased rate of re-epithelization and accelerated wound closure compared to control, no significant difference in efficacy between the CM and the cells has been demonstrated (Payne et al., 2010; Shohara et al., 2012; Shrestha et al., 2013; Aguilera et al., 2014; Raj et al., 2019; Zhang et al., 2020). Only rare studies used multiple doses of CM (Payne et al., 2010; Sun et al., 2019). It was observed that frequent application of CM achieved better results on wound healing than 2-fold application (Payne et al., 2010)**.**

#### Perinatal Cell-Derived Small Extracellular Vesicles (sEV)

The least used PnD type (9.6%) were perinatal cell-derived EV (Table 3). The cell secretome generally contains a mixture of different EV subtypes, including exosomes, apoptotic bodies and microparticles (Gangoda et al., 2015). The exosomes are the most characterized and the smallest EV. They are of endosomal origin with the size of 30–150 nm in diameter and carry many different components, such as lipids, proteins, mRNAs, non-coding RNAs, and even DNA derived from cells (reviewed in Rashed et al. (2017)). It was first thought that exosomes only play a role in removing unnecessary molecules from cells, but nowadays their involvement in a plethora of different cell responses is documented (Keller et al., 2006). According to their shape, size, and marker expression (HSP70, CD9, CD63, CD81), the EV described in the processed literature were characterized as exosomes. However, the International Society for Extracellular Vesicles in their position statement from 2018 (van Deun et al., 2017; Théry et al., 2018) urge authors to use an operational term for EV subtypes unless they can unequivocally prove their endosomal origin (by live imaging techniques, for example). Therefore, according to these recommendations, in this review we will refer to these EV as small EV, or sEV. The sEV derived from perinatal cells are a relatively new PnD type used for skin wound healing since they first appeared in publications only a few years ago. All included studies used sEV derived from MSC, most commonly from hUC-MSC (77.8%).

The sEV were applied mostly by subcutaneous injection in animals with burn or full-thickness wounds. The doses of sEV were predominantly expressed as μg/cm^2^, except for one study where the authors used the number of particles as a measure for the sEV quantity (Bian et al., 2020). The doses were comparatively uniform throughout the analyzed studies, mainly using 100 to 156 μg/cm^2^ (Zhang et al., 2015a; Zhang et al., 2015b; Zhang et al., 2016; Yang et al., 2019; Yang et al., 2020; Zhao et al., 2020) and 318 μg/cm^2^ in one study (Liu et al., 2021). There were no studies that compared different dosages of sEV.

Perinatal cell-derived sEV showed similar (Zhang et al., 2015a) or even better (Zhao et al., 2020) beneficial effects on wound healing compared to perinatal cells. Gao et al. (2020) observed that sEV overexpressing micro RNA (miR)-135a significantly accelerated fibroblast cell migration by downregulating LATS2 levels to promote wound healing in rats making it the first evidence of positive miRNA effect on this process. This was an interesting aspect, because so far, different miRNAs (including miR-135a) were mainly shown to impair wound healing (Bibby et al., 2021).

#### Perinatal Tissues

Perinatal tissues (of which hAM predominate with 85.7%) were applied in 15.1% of the included preclinical studies (Table 4). The hAM serves as a potential wound dressing due to its high biocompatibility, antimicrobial and anti-scarring properties. Its anti-inflammatory properties have been attributed to the decrease of pro-inflammatory cytokine expression (transforming growth factor ß and interleukin 10). Furthermore, it produces B defensins, elastase inhibitors, elastin, and lactoferrin with antimicrobial effects, making it highly attractive in healing leg ulcers (ElHeneidy et al., 2016). Decellularized or dehydrated perinatal tissues were used as grafts or as support for perinatal cells or cells of another origin and were applied topically. In cases where the entire full-thickness or burn wound area was covered with the hAM (Kim et al., 2009; Samadikuchaksaraei et al., 2016; Motamed et al., 2017; Song et al., 2017; Farzamfar et al., 2018; Gholipourmalekabadi et al., 2018; Kakabadze et al., 2019; Arasteh et al., 2020; Nasiry et al., 2020), the wound areas are given instead of dosages (Table 4). Also, in two studies where a proprietary human amnion/chorion graft (EpiFix^®^) was implanted subcutaneously, calculating the dosages was inapplicable (Koob et al., 2013; Koob et al., 2014).

It was shown that combinations of cells and decellularized hAM achieved better results on wound healing than either cells or hAM alone (Kim et al., 2009; Sabapathy et al., 2014; Samadikuchaksaraei et al., 2016; Motamed et al., 2017; Farzamfar et al., 2018; Gholipourmalekabadi et al., 2018; Kakabadze et al., 2019; Hashemi et al., 2020).

#### Perinatal Tissue Extracts

Of the included studies, 12.9% used perinatal tissue extracts (Table 5). The most frequently used tissue extract was hAM extract (41.7%). Tissue extracts are usually obtained by tissue lysis and centrifugation, hence they do not contain cells, but are rich in an array of proteins, minerals, amino acids, and steroid hormones (Datta and Bhattacharyy, 2012). Tissue extracts possess anti-inflammatory, antioxidant, and cytoprotective properties and stimulate proliferation and reparative processes similar to their tissues of origin (reviewed in Pogozhykh et al. (2018)).

Perinatal tissue extracts (some of which were prepared as a powder) were applied topically or by subcutaneous injection, mostly on full-thickness skin wounds, and once on either a burn wound or a skin flap model, respectively. The amount of the extracts used was usually expressed in μg of lyophilized powder or, in some cases, in μl of proprietary human placental extracts. The doses ranged from 1 μg to 83.3 μg/cm^2^. In two studies that compared the application of lower and higher doses of perinatal tissue extracts, higher doses performed better in terms of wound healing rate, reduced inflammation (Momeni et al., 2018), and skin flap survival (Kwon et al., 2019). Acellular umbilical cord-derived Wharton’s jelly extract (hWJ-ECM) was applied with Matrigel or in the form of a spongy scaffold in doses of 353.7 and 450 μl/cm^2^, respectively, providing enhanced wound closure and re-epithelization (Bakhtyar et al., 2017; Beiki et al., 2017). In one study, hybrid extracellular matrix sponges containing human placenta-derived extracellular matrix were applied within the wound area, but no volume or other quantitative value of the applied matrix was specified (Rameshbabu et al., 2018).

It was shown that amnion-derived hydrogel and amnion powder achieved better results on wound healing than AmnioGraft^®^ (Murphy et al., 2020). The combination of the human placental extract with autologous bone marrow MSC achieved better results on wound healing than placental extract applied without cells (Akela et al., 2013).

### *In vitro* Characterization and Functional Testing of PnD Before Their Application in Preclinical Studies

**FIGURE 3:**
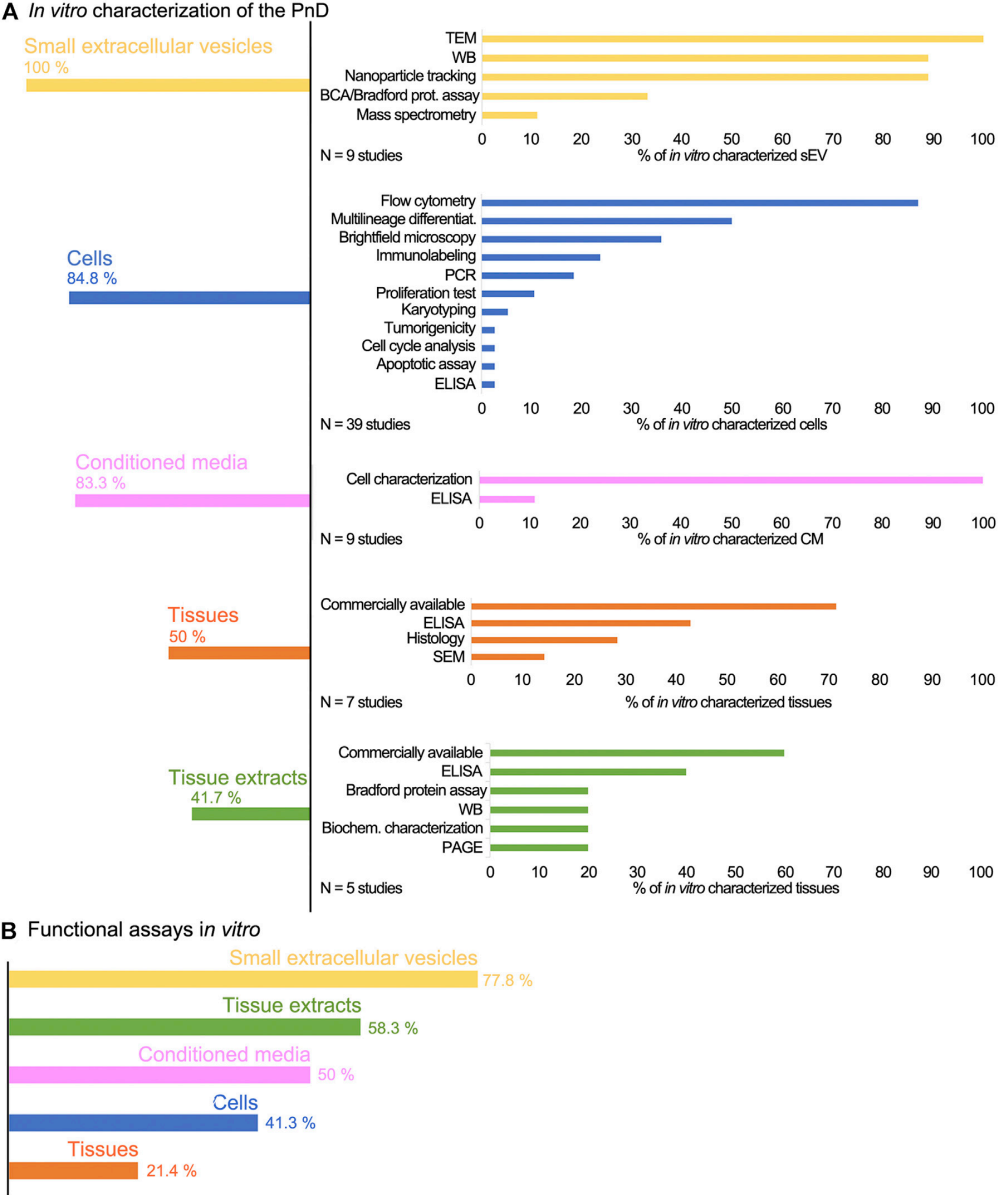
**(A)** The schematic presentation of the PnD characterization and **(B)** verification *in vitro*. The sEV derived from perinatal MSC are overall the most characterized and *in vitro* tested PnD used in skin wound healing in preclinical studies. Conversely, the least characterized and *in vitro* tested PnD are the perinatal tissues. The commercially available PnD or the PnD that have been described in the previous studies by the same authors, were defined as characterized *in vitro*, although the exact data were not provided in the actual study.

The evaluation of the *in vitro* characterization (Figure 3A) displayed the sEV as the most *in vitro* characterized PnD, as they were properly characterized in all included studies. The methods used for the characterization were: transmission electron microscopy to observe their morphology (100%), Western blot to determine the expression of exosome markers (89%), and nanoparticle tracking analysis to measure the size of sEV (89%). The reputable characterization of sEV is most probably due to the exact guidelines for isolation and characterization (van Deun et al., 2017; Théry et al., 2018). Encouraging the scientific community to properly perform exosome characterization has proven to be a good practice that provides transparency to the studies, and should also be considered in other fields. Cells were also fairly well characterized *in vitro* (84.8%). The predominant methods were: flow cytometry to identify the presence of the cell surface markers (87.2%), assessment of the differentiation potential into multiple lineages to confirm their stemness (50%), and bright field microscopy to verify morphological characteristics (35.9%). Of perinatal cell-derived CM, 83.3% were characterized *in vitro.* CM was biochemically analyzed in just one of the studies (Sabzevari et al., 2020), however, we further defined CM as “*in vitro* characterized” also when the corresponding cells were identified and characterized at the molecular and morphological levels. Perinatal tissues were *in vitro* characterized with a proportion of 50%; this group also includes commercially available perinatal tissues (71.4%).

We considered commercially obtained perinatal tissues as “*in vitro* characterized,” since the vendors clearly describe them. As the most commonly used perinatal tissue is the hAM, we assume that the poor percentage of the *in vitro* characterization is due to the fact that the hAM has been characterized and tested many times before. Further, identification and preparation of the hAM are unambiguous and straightforward (Jerman et al., 2014; Poženel et al., 2019; Jerman et al., 2020; Ramuta et al., 2020; Weidinger et al., 2020), whereas cells and cell-derived secretomes or sEV require precise characterization. Since the vast majority of the researchers isolated cells from the perinatal tissues by themselves, adequate characterization is mandatory to assure that a pure cell population has been isolated. The least *in vitro* characterized PnD were perinatal tissue extracts (41.7%).

In addition to *in vitro* characterization, *in vitro* functional tests were performed (Figure 3B). The differentiation and proliferation assays, scratch wound assay, and cytotoxicity assay were the most prevalently used to evaluate the effect of different PnD on cell cultures *in vitro*. The *in vitro* functional tests are usually quick and performed under controlled conditions. Compared to the animal models, they certainly lack the complexity of the native tissue. Nevertheless, monitoring the specific mechanisms *in vitro* is a good indicator of how the tested materials affect the biological processes *in vivo*. Among the PnD used in the preclinical studies of skin wound healing, 77.8% of the perinatal cell-derived sEV were functionally tested *in vitro*. Moreover, 58.3% of included studies performed functional tests *in vitro* on perinatal tissue extracts, and half of the studies functionally tested the perinatal cell-derived CM. On the other hand, despite being the most frequently used PnD in skin wound healing, only 41.3% of the perinatal cells were subjected to functional *in vitro* assays before their application in preclinical studies. The least *in vitro* tested PnD were perinatal tissues (21.3%).

## Animal Models of Cutaneous Wound Healin**g**

Animal models span various wound types, multiple animal species, and strategic scientific approaches for wound treatment. According to most common problems in wound healing in humans, such as full-thickness wounds, chronic ulcers caused by diabetes, poorly healing wounds after radiation, or severe burn injuries, the animal models are adapted to these conditions as well as possible.

**FIGURE 4:**
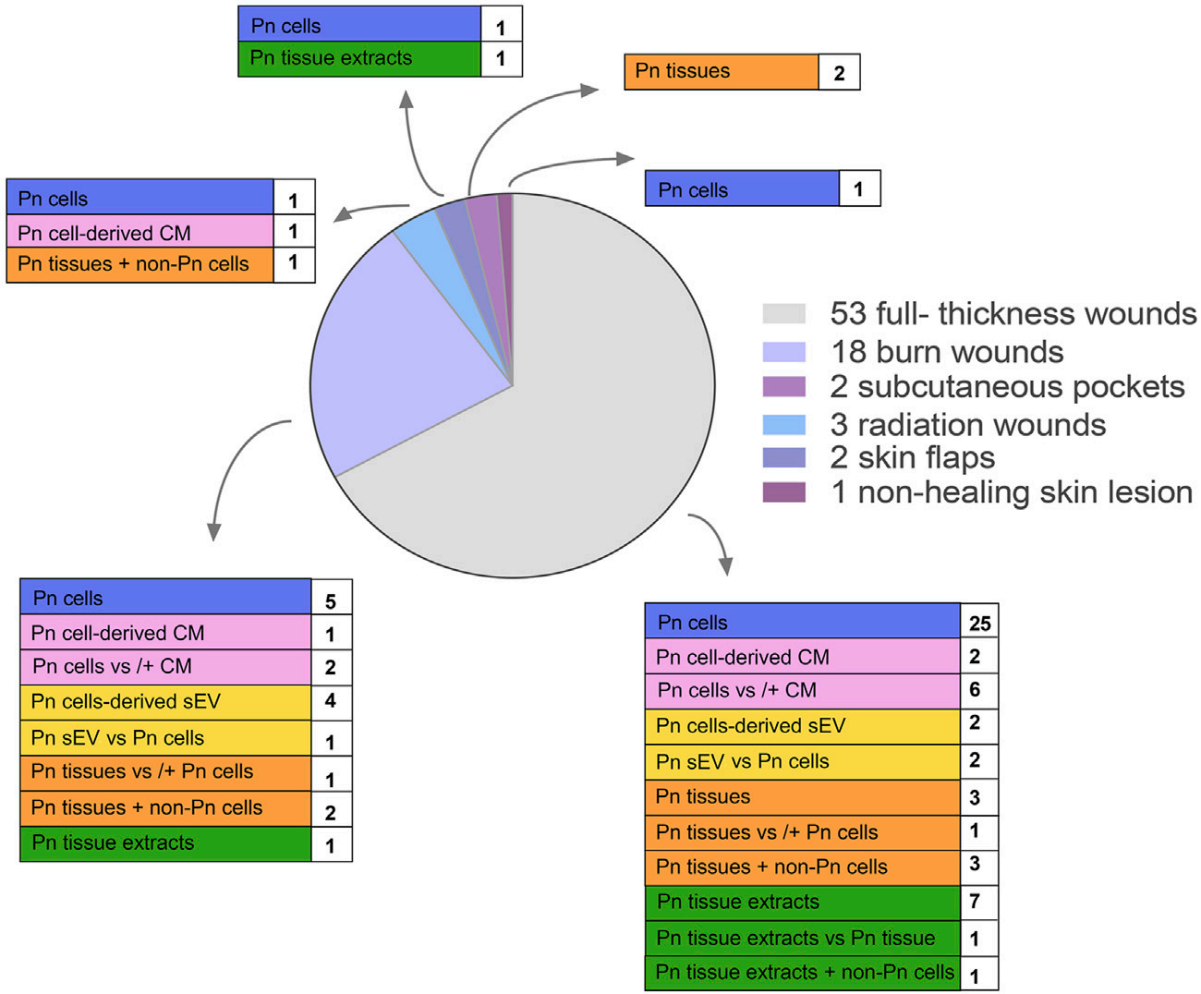
Distribution of wound types and applied PnD types.

The herein described animal models dealt with full-thickness wounds (53 studies), burn wounds (18 studies), radiation wounds (three studies), skin flaps (two studies), and subcutaneous pockets (two studies). One case report described the application of PnD on non-healing skin lesions (Figure 4; Tables 1–5).

### Wound Models

#### Full-Thickness Wound Models

In full-thickness wounds, all skin layers including the subcutaneous tissue, are removed. Full-thickness wounds were inflicted mainly by biopsy punches or by scalpels and surgical scissors. In addition to healthy animals, this wound type has also been applied to diabetic animals to mimic impaired healing conditions and chronic wounds.

#### Diabetic Wound Models

Diabetes is a major health care problem. A diabetes-related foot ulcer is one of the most challenging complications in the treatment of diabetic patients. More than half of diabetic ulcers become infected and lead to amputation in 20% of cases. (Armstrong et al., 2017). Thus, several studies use diabetic mouse and rat models to develop new strategies for treatment.

Experimental type 1 diabetes can be studied on a genetic non-obese diabetic mouse line or on mice/rats treated with streptozotocin, which destroys pancreatic beta cells. The advantage of diabetes induced by streptozotocin injection is that there is no limitation to a particular mouse or rat strain. Two studies used the non-obese diabetic/severe combined immunodeficiency (NOD/SCID) mice, which have a metabolic state similar to type 1 diabetes ((Jin et al., 2016) and (Kim et al., 2012)). One study used a streptozotocin-induced C57BL/6J mouse (Zhang et al., 2020). Four studies used diabetic rat models on streptozotocin-induced SD rats (Han et al., 2019; Shi et al., 2020; Yang et al., 2020; Yue et al., 2020). One further study used streptozotocin-induced Wistar rats (Milan et al., 2016).

Type 2 diabetes can be induced by a high-fat diet or genetically in the leptin-deficient mouse line (Boyko et al., 2017). Four studies (Shrestha et al., 2013; Peña-Villalobos et al., 2018; Raj et al., 2019; Bian et al., 2020) used leptin-deficient db/db mice as a diabetes type 2 model. Affected mice show morbid obesity, chronic hyperglycemia, pancreatic beta-cell atrophy, and hypoinsulinemia. They are polyphagic, polydipsic, and polyuric, similar to diabetic type 2 patients. This mouse line is recommended for wound healing models^2^.

Wang et al. (2016) used the Goto-Kakizaki rats as a non-obese model of type 2 diabetes with metabolic, hormonal, and vascular disorders similar to human diabetes.

#### Burn Injury Wound Models

Burn wounds can be created by scald, contact, chemical or electrical combustion. The severity of the burn wound (Supplementary Table S1) depends on the temperature and exposure time of the heat source to the skin, as well as on the skin thickness.

Except for the study on pigs (Eylert et al., 2021), all burn injury wound models of the reviewed studies were performed on rodents (primarily mice and rats, with one study was performed on guinea pigs). Burn wounds were created by scalding with hot water (70°C–94°C) for 6–100 s (Liu et al., 2014; Zhang et al., 2015a; Zhang et al., 2015b; Du et al., 2016; Liu et al., 2016; Zhang et al., 2016; Momeni et al., 2018; Li et al., 2019; Yang et al., 2019; Liu et al., 2021) or by a heated brass rod or aluminum devices (100–105°C, for 5–30 s), where wound size and shape depend on the size and shape of the instrument used (Motamed et al., 2017; Gholipourmalekabadi et al., 2018; Pourfath et al., 2018; Mahmood et al., 2019; Rahman et al., 2019; Hashemi et al., 2020). As wounding by the brass bar technique leads to scab formation, the developed necrosis was usually excised 12–48 h before application of PnD.

In most studies, severe burn injuries of 2nd or 3rd degree were performed. Some authors described the injury as “full-thickness burn injuries” which would correspond to at least category IIa according to the severity score. In cases with extensive wounds, an injection of balanced salt solution was given to prevent shock.

Uniform partial-thickness burns cannot be reproducibly created in mice and rats due to their estrous hair cycle (Robson et al., 1980), and superficial burns in pigs heal too quickly, because the porcine epidermis is 2-fold thicker than the human equivalent (Hammond, 2000). Thus, a higher temperature is needed to create severe burn wounds in pigs. (Eylert et al., 2021) used a heated aluminum device (200°C) for 20s and digital force gauge and histologically confirmed full-thickness burn wounds 48 h post-burn via punch-biopsy. Payne et al. (2010) studied partial-thickness burn wounds on guinea pigs, which do not have hair cycles and are therefore the more satisfactory model to evaluate this wound type. Only one study investigated superficial first-degree burn wounds on rats (Momeni et al., 2018).

Some authors described the percentage of the total body surface area (%TBSA) affected by a burn. 10% TBSA wounds were performed on guinea pigs (Payne et al., 2010), 30% TBSA and 50% TBSA on rats (Liu et al., 2014; Liu et al., 2016).

Due to a large number of different parameters such as wound size, heat exposure time, application of brass bar or water, inconsistent use of the severity score, and different treatments and application modes with PnD, a comparison of investigated experiments is not possible.

#### Radiation-Induced Wound Models

Radiation dermatitis is a common side effect of radiotherapy. Many of the radiation-induced skin changes are minor and reversible. Nevertheless, when acute changes do not resolve, skin ulcers, fibrosis, or necrosis of underlying structures may occur. Disruption of the epithelial basement membrane and breakdown of the barrier function substantially increase the risk for these injuries (Hymes et al., 2006). The severity grade is shown in Supplementary Table S2 according to the Classification of radiation dermatitis from the National Cancer Institute (Hymes et al., 2006).

In the studies reviewed, 3rd or 4th-grade radiation wounds were created in rats (Liu et al., 2014; Kakabadze et al., 2019; Sun et al., 2019), respectively. Radiation dose and exposure time varied between the different studies. Liu et al. (2014) administered a dose of 45 Gy for 7.5 min per animal, Sun et al. (2019) administered 40 Gy for 25 min and Kakabadze et al. (2019) used a dose of 60 Gy without specifying of the radiation duration.

Sun et al. (2019) topically applied Hydrogel containing hUC-MSC-CM every second day after radiation for 8 weeks. PnD treatment accelerated wound closure, sebaceous gland regeneration, and angiogenesis compared to EGF application and negative control (weeks 2, 4, 6, 8).

Liu et al. (2014) created ulcers and Kakabadze et al. (2019) excised the radiation areas 3 weeks post-radiation to simulate a chronic radiation injury before starting the treatment with PnD. Subcutaneous injection of hUC-MSC increased neovascularization and re-epithelization, days 14, 21, 28 after treatment (Liu et al., 2018). Topical application of decellularized hAM seeded with BM-MSC (Kakabadze et al., 2019) accelerated wound closure compared to decellularized hAM. Inflammation and exudations were absent when the decellularized hAM was used (observation period 90 days).

Due to different intervals of evaluation, only days 7 and 14 could be used for a comparison, whereby the comparison is limited by different doses of radiation, different surgical interventions post-radiation, and different time points of application of the various PnD. In all investigated works, wounds treated with PnD showed a significantly accelerated wound healing on day 14 compared to control groups.

Due to the limited data and varying methods, a clear recommendation for the optimal treatment method concerning the application of PnD for the healing of radiation-induced wounds is not possible.

#### Skin Flap Models

A skin flap is a full-thickness mass of skin containing superficial fascia, transplanted from a donor site to a recipient site with an intact blood supply. Skin flap surgery is a common procedure in reconstructive surgery. In this field, surgeons often struggle with ischemia-associated complications such as tissue necrosis or wound breakdown (Schmauss et al., 2018). Flap models in rodents are versatile and have a long tradition in experimental surgery. In studies included in this review, two types of skin flaps were performed: the epigastric ischemic skin flap (EIF) and the McFarlane flap.

The EIF is an axial skin flap, designed at the pedicled superficial inferior epigastric vessels. The skin flap is lifted from its remote end, and the vascular pedicle gets clamped for a specific time to induce flap ischemia. Leng et al. (Leng et al., 2012) performed the EIF on mice at a size of 3 × 6 cm and clamped the respective blood vessels for 6 h. Then, the flap was opened to remove the clamps and to enable flap reperfusion. The flap was sutured *in situ* and hUC-MSC were subcutaneously injected into the flap at 10 distributed points. After 7 days, the survival area of the flap was evaluated concerning the gross appearance, necrosis, and vascularization. hUC-MSC were detectable in the flap tissues and increased the survival of the flap, neovascularization, and expression of bFGF and VEGF compared to controls without cell treatment.

The McFarlane flap model is characterized by a cranially based and randomly perfused dorsal skin flap, which is elevated beneath the musculus panniculus carnosus. It has a defined width-to-length ratio to ensure a predictable rate of necrosis. Perforating blood vessels were electrically cauterized to ensure a completely random vascular pattern. Kwon et al. (2019) performed this model on rats with a flap size of 3 × 10 cm to investigate therapeutical effects of human placenta extract (hP-E) applied by subcutaneous or intraperitoneal injections. hP-E enhanced flap survival, angiogenesis, reduced necrotic areas, induced antioxidative response, and inhibited apoptosis compared to phosphate-buffered solution (PBS) control. Daily application (d0-d6) of low dose (10 mg/kg/d) localized or systemic hP-E injections or high dose (40 mg/kg/d) systemic hP-E injections showed the best effects of high dose administration (d7).

Although data on using PnD in skin flap models are rare, both approaches showed promising results.

#### Subcutaneous Pocket Model

Koob et al. (2013), Koob et al. (2014) created subcutaneous pockets in mice and implanted commercially available dehydrated human amnion/chorion-tissue allografts (Purion^R^) at a size of 5 × 5 mm to investigate their biological and angiogenic properties. hAM/chorion implants recruited more mesenchymal progenitor cells to the site of implantation compared to uninjured skin and the sham implant site (day 7). In addition, implants displayed a steady increase in microvessels approaching that of healthy and healing skin after 28 days.

### Animal Species Used for Cutaneous Wound Healing Models

#### Rodents

The vast majority of animal wound models (92%) were performed on rodents, with 40 and 32 studies performed on mice and rats, respectively. Only one study was performed on guinea pigs. Rodents are easy to handle and inexpensive with respect to costs for food, medication, and wound dressings. They are simple to house and have a short life cycle. Generally, smaller wounds were created on mice and rats than on larger animals, which helps to reduce the quantity of applied PnD material. However, rodents’ wounds close due to the contraction of the *musculus panniculus carnosus*, a thin layer of skeletal muscle located in subcutaneous tissue, which is virtually non-existent in most regions of the human body (Zomer and Trentin, 2018). This physiological difference creates difficulties in replicating the wound closure processes of human skin, which should be considered. For long-time experiments on full-thickness wounds, anti-contractive tools should be chosen (described in the chapter *Wound Healing in Rodents*).

##### Mouse Strains Used in Wound Healing Studies

**FIGURE 5:**
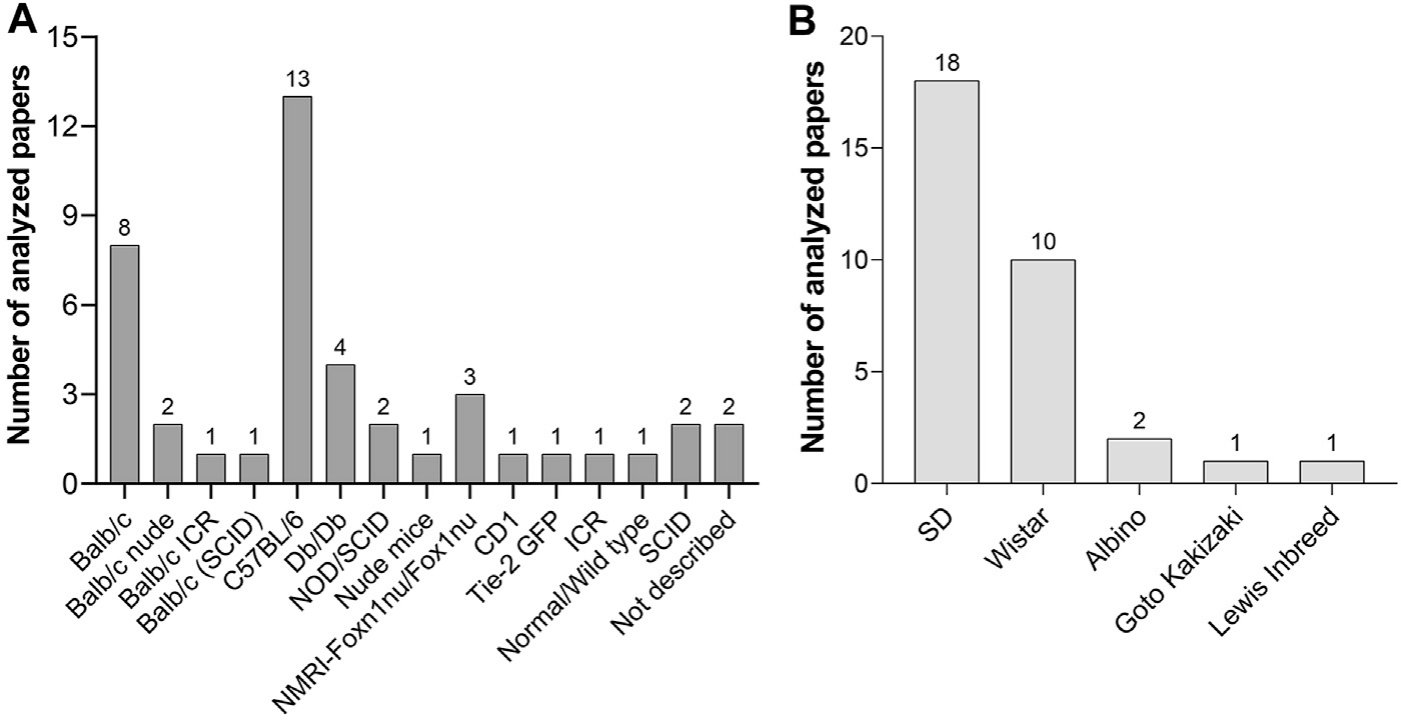
**(A)** Mouse strains and **(B)** rat strains used for wound healing experiments with PnD treatment in scientific papers published between 2004 and 2020.

The C57BL/6 mouse was the most commonly used strain in the analyzed studies (Figure 5A). It is a general-purpose strain. Some of its characteristics are a high susceptibility to diet-induced obesity, diabetes II, and atherosclerosis. It has a nearly black coat, is easy to breed, and is robust. However, it tends to bite, and male mice remove hair from their cage mates (Sarna, 2000). Its barbering behavior may be counterproductive for an undisturbed wound healing process, as these animals might nibble on wound dressings. Most importantly, these animals are unusually sensitive to pain and cold, and analgesic medications are less effective (Mogil et al., 1999). Thus, the selection of C57BL/6 mice for wound healing experiments needs to be carefully evaluated.

Another commonly used mouse type was the albino Balb/c strain with its different inbreeds (Balb/c ICR, Balb/c (SCID), and Balb/c nude). BALB/c mice are more docile than C57BL/6 mice and are particularly well known for the production of monoclonal antibodies. BALB/c mice are used in many research fields (cardiovascular research, cancer, infectious diseases, neurobiology immunology, inflammation, autoimmunity). As most substrains display high levels of anxiety, (Griebel et al., 1993; Belzung and Berton, 1997) a familiar, calm environment is recommended.

The ICR inbred strain serves as a general-purpose strain and does not develop insulitis or diabetes. These mice were recommended amongst others for drug testing and as a control for non-obese diabetic mice^3^.

CD1 are albino mice and are suggested as a general multipurpose model, for safety and efficacy testing, as an aging, pseudopregnancy, and surgical model^4^.

*SCID* Mice are homozygous for the severe combined immune deficiency spontaneous mutation (*Prkdc* ^*scid*^). They are characterized by an absence of functional T cells and B cells, and a normal hematopoietic microenvironment. As *SCID mice* accept allogeneic and xenogeneic grafts, they are an ideal model for cell transfer experiments. Immunodeficient mice should be housed in a specific-pathogen-free environment to avoid infections^5^.

Nude mice such as the Balb/c nude or the NMRI-Foxn1nu/Foxn1nu inbred are characterized by thymic aplasia, which results in immunodeficiency due to the lack of T cells. They have no rejection responses. The mutation leads to a keratinization defect of the hair follicles and the epidermis^6^. The mostly hairless phenotype helps to avoid a shaving procedure before the wound healing experiments. They are sensitive to cold and need to be kept at warm temperature conditions.

TIE-2 GFP (B6.Cg-Tg287Sato/1) strain expresses Green Fluorescent Protein under the direction of the endothelial-specific receptor tyrosine kinase (*Tek*, formerly, *Tie2*) promoter. This mouse type is especially appropriate to study neovascularisation during the wound healing process as GFP-expressing endothelial cells can be visualized via fluorescent microscopy (Wang et al., 2018).

##### Rat Strains Used in Wound Healing Studies

Compared to mouse strains, fewer rat strains were used in the investigated wound healing experiments (Figure 5B). Sprague Dawley (SD) and Wistar rats are the most popular albino rats used for laboratory research. SD rats are calm and less active than Wistar rats and are therefore easy to handle. Lewis rats are highly sensitive to the induction of autoimmune diseases as well as to diet-induced obesity and diabetes and streptozotocin-induced diabetes^7^. The Goto-Kakizaki inbred line was used as a non-obese model of type 2 diabetes.

##### Wound Healing in Rodents

In general, larger wounds were created on rats than on mice (one to two full-thickness wounds/animal with an average size of 1.52 vs. 2.11 cm^2^ and one to two burn wounds/animal with an average size of 1.37 vs. 3.8 cm^2^ in mice and rats, respectively. Larger wounds were radiation wounds created on rats (8 cm^2^) and skin flaps (18 cm^2^ on mice, 30 cm^2^ on rats). Several approaches were developed to get similar conditions to human wound healing through the generation of granulation tissue and re-epithelialization rather than contraction of the *musculus panniculus carnosus*. One possibility is to investigate early stages of wound healing in short-term studies lasting up to 8 days post wounding, where no apparent wound contraction occurs (as performed by Tuca et al. (2016), Ertl et al. (2018)).

For longer-term experiments on full-thickness wounds, anti-contractive tools should be chosen: Splinting wound models use silicone or rigid plastic rings strapped around the wound area to the underlying muscles to prevent wound contraction. In the reviewed papers, the silicone rings were mostly sutured or fixed with glue (Kim et al., 2012; Shohara et al., 2012; Aguilera et al., 2014; Jin et al., 2016; Wang et al., 2016; Peña-Villalobos et al., 2018; Wang et al., 2018; Kaushik and Das, 2019; Arasteh et al., 2020; Nasiry et al., 2020; Sabzevari et al., 2020). Others directly sutured scaffolds loaded with PnD to the wound site (Yang et al., 2013; Edwards et al., 2014; Milan et al., 2016; Montanucci et al., 2017). Edwards et al. (2014) additionally placed a titanized mesh between the wound bed and a collagen-based scaffold (loaded with hUC-MSC) to avoid tissue contraction. However, these methods carry risks of inflammation and surgical site infection (Mulholland, 2020). Some studies did not use suturing of the scaffolds (or at least did not mention suturing) (Nan et al., 2015; Du et al., 2016; Han et al., 2019; Raj et al., 2019).

Beiki et al. (2017) observed that scaffolds produced from liquid WJ-ECM accelerated wound closing and counteract wound contracture during the observation period of 18 days. Also, a hyaluronic acid hydrogel with or without solubilized hAM counteracted wound contracture (Murphy et al., 2017). We could see from Vonbrunn et al. (2020) that the application of stiff carrier materials for PnD such as electrospun Poly(ε-caprolactone)/poly(l-lactide) (PCL/PLA) could enable uncomplicated experiments on rodents without suturing and could open new therapeutic approaches due to the anti-contractive properties of the material. Still, numerous long term-studies were performed on rodents, where no anti-contractive strategies were applied. This hinders the reproducibility of data, and the studies’ outcomes seem questionable. Anti-contractive properties are strongly needed, especially if PnD were just subcutaneously injected around the wound area or are topically applied in fluid or gel-like solutions or as soft membranes.

**FIGURE 6:**
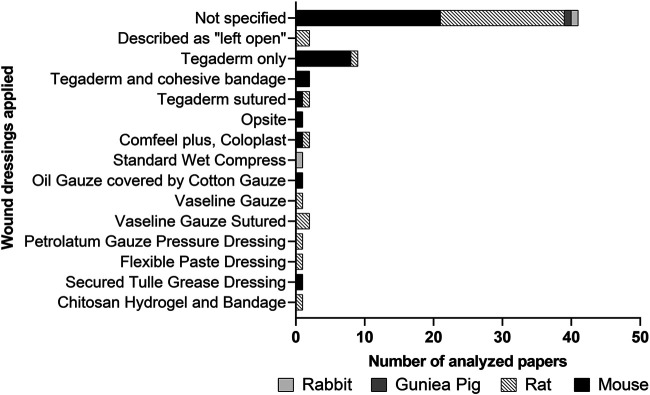
Types of wound dressing applied to animal cutaneous wounds.

Another problem for the reproducibility of data is that there is significant variability in the application of wound dressings. Wound dressings are used to keep the wound area free of contamination. In the majority of the analyzed studies on rodents, it is not described whether a wound dressing was applied or not (Figure 6). Thus, it is difficult to evaluate the wound healing progress under unspecified experimental settings. In two studies, wounds were left open (Liu et al., 2014; Liu et al., 2016).

A popular wound dressing material for rodents is a dressing with Tegaderm™ plaster. It was applied without suturing (Zebardast et al., 2010; Tuca et al., 2016; Ertl et al., 2018; Bian et al., 2020; Vonbrunn et al., 2020), sutured onto the skin (Eylert et al., 2021), or was combined with a cohesive bandage (Arasteh et al., 2020). Other possibilities for wound dressings are fat gauze or oil gauze – with and without suturing to the skin (Montanucci et al., 2017; Motamed et al., 2017; Song et al., 2017; Gao et al., 2020; Hashemi et al., 2020; Zhao et al., 2020).

As unsutured plasters were partly detached from the wounds after a few days, we recommend removing the plasters 3 days post wounding, and thereafter leaving the wounds open to get standardized conditions for the wound healing process.

An accurate description of the advantages or disadvantages of used wound dressings in future publications would be helpful to standardize experiments and to enable comparability of the experiments concerning the healing outcome.

#### Wound Healing in Large Animals

On large animals, experiments were mainly performed on pigs. Pigs have the advantage that the porcine skin more closely correlates to human skin in thickness and structure than rodent skin. Wound closure in pigs is similar to wound healing in humans because the *musculus panniculus carnosus* is vestigial or absent in most body regions. Therefore, wounds heal by re-epithelialization rather than by contraction, and no anti-contractive strategies or devices are needed. However, experiments on pigs are cost-intensive and far more complex with respect to medication, anesthesia, and wound dressing, making them mainly useful for preclinical trials of therapies (Boyko et al., 2017).

As the number of animals in experiments is limited due to costs and husbandry, multiple wounds were performed on each subject.

Three studies included in the review utilized pigs as experimental animals (Han et al., 2014; Murphy et al., 2020; Eylert et al., 2021). Multiple wounds were created on the back of the animals (minimum 4, maximum not specified) and topically treated with PnD for 3–4 weeks. (Eylert et al., 2021) performed multiple full-thickness burn wounds with a size of 5 × 5 cm in diameter. It was shown that wounds treated with Integra^®^ seeded with hUC-MSC at a low dose (40,000 cells/cm^2^) regenerated wounds most efficaciously. Wounds were dressed with a layer of topical antibiotics, fat gauze, multiple layers of gauze, Tegaderm^®^, and a compression jacket. The initial wound margins were marked by a skin stapler. Wound dressing changes were performed two to three times per week.

Murphy et al. (2020) created eight 4 × 4 cm square-shaped full-thickness wounds on the central back and marked initial wound margins by tattooing before excision. Amnion-hydrogel and amnion powder treatments achieved the most rapid wound healing compared to commercially available Amniograft^®^, followed by hydrogel only, untreated wounds, or graft jacket, respectively. The treatment with the graft jacket alone led to the worst healing outcome (largest wound area, most contraction, least epithelialization). The wound dressing consisted of a topical antibiotic cream, Tegaderm^®^ and a cast padding, cohesive bandaging, a protective saddle, and a jacket.

Han et al., 2014 (Han et al., 2014) created circular full-thickness wounds (diameter 3 cm). Collagen-chitosan-based scaffolds loaded with microencapsulated VEGF gene-modified hUC-MSC improved the vascularization of the tissue-engineered dermis and induced a better wound healing than controls (microencapsulated hUC-MSC, empty capsule, and PBS-treated group). The wound dressing was not described.

One case report described the wound treatment on dogs (Ribeiro et al., 2014). Two dogs suffering from non-healing skin lesions were treated with poly(vinyl alcohol) hydrogel (PVA) membrane supplied with low-dose UC-MSC 0.1 × 10^6 cells/cm^2^. hUC-MSC induced significant progress in skin regeneration with the decreased extent of ulcerated areas.

Two studies were performed on rabbits, where full-thickness wounds were treated with rabbit BM-MSC either in combination with hAM (Kim et al., 2009) or with human placenta extract (Akela et al., 2013).

## Summary and Conclusion

The pre-clinical evidence of our review indicates that PnD-based therapy is in general effective to promote cutaneous wound healing. The significant amount of variability between types, dosage, and application mode of PnD, as well as between the animal models, hinders the comparability of data. Perinatal cells were the most commonly used PnD type, but only a tiny minority of studies compared the effect of applying different cell concentrations or modes of administration. Perinatal cell treatment worked at both low and high dosing, and further studies are required to evaluate the most optimal cell therapy. Treatment with perinatal cell-derived CM achieved similar beneficial effects as with cells, and frequent multiple applications of CM showed better outcomes than single- or two-fold treatments. Perinatal cell-derived sEV showed similar or even better beneficial effects on wound healing than perinatal cells. No studies compared different dosages of perinatal cell-sEV. The combination of cells and perinatal tissue membranes achieved better results on wound healing than either cells or tissue alone, and the combination of perinatal tissue extracts with cells achieved better results than tissue extracts without cells.

As an inadequate characterization of PnD may lead to a false identification of the PnD that hinders reproducibility and comparability of various studies, we propose the following guidelines for the PnD characterization before their application in pre-clinical studies:

1) A detailed and traceable cell/tissue isolation procedure – if PnD are commercially available, the company should be indicated (catalog and lot numbers, etc.).
2) Each PnD should be characterized by at least two *in vitro* methods: For cells, this should include the phenotype (morphology, immunolabeling for mesenchymal/epithelial markers, stem cell markers) and the ability to differentiate into all three germ layers (if stem cells are used). For the tissues, histology and immunolabeling to prove the identity/characteristics of the PnD, should be provided. In the case of purchased cells/tissues, the authors should indicate the main characteristics of PnD, as provided by the company.
3) Functional tests on cytotoxicity, promotion of proliferation, migration, and differentiation should be performed to ensure the quality of the PnD.
4) Dosage of PnD, characteristics of the wound, and the entire operating procedure, with mode and timing of application, should be described in detail so that the methodology is fully reproducible.

In addition, well-defined and standardized animal models are required for a comparison of outcome measures between studies. They may help to minimize redundancy in animal experimentation in line with the Replacement, Reduction, and Refinement (3R) principles for more ethical use of animals in research (Russell and Burch, 1960). The needs for standardization range from the choice of animal species and strains (with a preference for docile strains without barbering behavior and low anxiety levels) to precise experimental settings with regard to the extent and deepness of wounding and the adequate wound dressing. On rodents, anti-contractive strategies, ideally without suturing of devices, should be considered for long-term experiments (longer than 1 week) on full-thickness wounds.

An accurate description of the advantages or disadvantages of methods used in future publications would be helpful to standardize experiments and to enable comparability of the experiments concerning the healing outcome.

In conclusion, PnD have a promising potential to be widely used as a source of biological material to assist wound healing, now and in the future. Further concerted actions will be needed to bridge the gap between PnD basic research, pre-clinical studies, and their translation into the clinic. The COST SPRINT Action (CA17116) aims to provide comprehensive and evidence-based guidelines on all levels (Silini et al., 2020) to promote the safety and efficacy of the therapeutic use of PnD. The present review will contribute to the establishment of standards of care in wound healing.

## Data Availability

The original contributions presented in the study are included in the article/Supplementary Material, further inquiries can be directed to the corresponding author.

## Footnotes

1
https://clinicaltrials.gov/

2
https://www.criver.com/products-services/find-model/jax-dbdb-mice?region=3616/

3
https://www.jax.org/strain/009122

4
https://www.criver.com/products-services/find-model/cd-1r-igs-mouse?region=3616

5
https://www.criver.com/products-services/research-models-services/animal-models/mice/immunodeficient-mice/scid-mice?region=3616

6
https://www.jax.org/

7
https://www.janvier-labs.com/en/fiche_produit/lewis_rat/
