# Supplementary material for: Systematic Review of the Application of Perinatal Derivatives in Animal Models on Cutaneous Wound Healing
Source: Front Bioeng Biotechnol. 2021 Sep 24;9:742858. doi: 10.3389/fbioe.2021.742858 (PMC8498585; doi:10.3389/fbioe.2021.742858)
Supplement: Supplementary file 1 [file Table1.DOCX]

Supplementary Material

# Supplementary Boolean Search string for data analysis

(placenta OR placental OR "perinatal tissue" OR “neonatal tissue” OR decidua OR amnion OR “amniotic fluid” OR "amniotic membrane-derived” OR "human amniotic membrane" OR "umbilical cord" OR "Wharton's jelly" OR "Wharton jelly" OR "Whartons jelly" OR chorion OR "chorionic membrane" OR "fetal membrane" OR “fetal tissue” OR "villous stroma") AND ("stem cells" OR "progenitor cells" OR "stroma cells" OR "stromal cells" OR "mesenchymal cells" OR "amnion epithelial cells" OR "amniotic epithelial cells" OR "amniotic membrane-derived cells" OR "amniotic membrane transplantation" OR “extracellular vesicles” OR exosomes OR microvesicles OR secretome OR “conditioned medium” OR scaffold OR “protein extracts” OR “extract”) NOT ("umbilical cord blood" OR “cord blood” OR hematopoietic OR haematopoietic OR review[Publication Type]) AND (animal OR "in vivo" OR preclinical OR pre-clinical OR mouse OR mice OR rat OR rodent OR rabbit OR sheep OR ovine OR swine OR pig OR horse OR equine OR cow OR bovine OR dog OR canine OR fish OR primate OR primates OR organoids OR “decellularized matrix” OR “de-cellularized matrix” OR “decellularised matrix” OR “de-cellularised matrix”)

# Supplementary Table 1: Severity grade of burn wounds

| Degree |  |
| --- | --- |
| I | Limited to the epidermis. Erythema, tightness, touch sensitive, scar less healing. |
| IIa | Damage of the epidermis and superficial parts of the dermis. Erythema, development of blisters, risk of infection, hair roots, glands and skin receptors intact, due to that painful. No development of scars, possibility of pigment disorder. |
| IIb | Damage of epidermis and deep parts of the dermis. Development of blisters, humid to dry wound bed, Increased texture and whitish to reddened areas. Due to damage of pain- and tactile receptors loss of sensibility and reduction of sensation of pain. Wound healing with scar formation. |
| III | Damage of all skin layers including the subcutis. Greyish to yellowish wax-like discolorations of skin with visible thrombosed blood vessels. Loss of hair and nails, fully loss of tactile and pain receptors. Wound healing with scar formation and development of keloid. |

# Supplementary Table 2: Severity grade of radiation induced wounds

| Grade |  |
| --- | --- |
| 0 | None |
| 1 | Faint erythema or dry desquamation |
| 2 | Moderate to brisk erythema or patchy moist desquamation, mostly confined to skin folds and creases; moderate edema |
| 3 | Confluent moist desquamation, > 1.5 cm diameter, not confined to skin folds: pitting edema |
| 4 | Skin necrosis or ulceration of full-thickness dermis; may include bleeding not induced by minor trauma or abrasion |
